# A visual and curatorial approach to clinical variant prioritization and disease gene discovery in genome-wide diagnostics

**DOI:** 10.1186/s13073-016-0261-8

**Published:** 2016-02-02

**Authors:** Regis A. James, Ian M. Campbell, Edward S. Chen, Philip M. Boone, Mitchell A. Rao, Matthew N. Bainbridge, James R. Lupski, Yaping Yang, Christine M. Eng, Jennifer E. Posey, Chad A. Shaw

**Affiliations:** Program in Structural and Computational Biology and Molecular Biophysics, Baylor College of Medicine, Houston, TX 77030 USA; Department of Molecular & Human Genetics, Baylor College of Medicine, Houston, TX USA; Human Genome Sequencing Center, Baylor College of Medicine, Houston, TX USA; Department of Pediatrics, Baylor College of Medicine, Houston, TX USA; Department of Pediatrics, Texas Children’s Hospital, Houston, TX USA; Baylor Miraca Genetics Laboratories, Baylor College of Medicine, Houston, TX USA; Department of Statistics, Rice University, Houston, TX 77005 USA

**Keywords:** Disease gene discovery, Exome, Semantic similarity, Variant prioritization

## Abstract

**Background:**

Genome-wide data are increasingly important in the clinical evaluation of human disease. However, the large number of variants observed in individual patients challenges the efficiency and accuracy of diagnostic review. Recent work has shown that systematic integration of clinical phenotype data with genotype information can improve diagnostic workflows and prioritization of filtered rare variants. We have developed visually interactive, analytically transparent analysis software that leverages existing disease catalogs, such as the Online Mendelian Inheritance in Man database (OMIM) and the Human Phenotype Ontology (HPO), to integrate patient phenotype and variant data into ranked diagnostic alternatives.

**Methods:**

Our tool, “OMIM Explorer” (http://www.omimexplorer.com), extends the biomedical application of semantic similarity methods beyond those reported in previous studies. The tool also provides a simple interface for translating free-text clinical notes into HPO terms, enabling clinical providers and geneticists to contribute phenotypes to the diagnostic process. The visual approach uses semantic similarity with multidimensional scaling to collapse high-dimensional phenotype and genotype data from an individual into a graphical format that contextualizes the patient within a low-dimensional disease map. The map proposes a differential diagnosis and algorithmically suggests potential alternatives for phenotype queries—in essence, generating a computationally assisted differential diagnosis informed by the individual’s personal genome. Visual interactivity allows the user to filter and update variant rankings by interacting with intermediate results. The tool also implements an adaptive approach for disease gene discovery based on patient phenotypes.

**Results:**

We retrospectively analyzed pilot cohort data from the Baylor Miraca Genetics Laboratory, demonstrating performance of the tool and workflow in the re-analysis of clinical exomes. Our tool assigned to clinically reported variants a median rank of 2, placing causal variants in the top 1 % of filtered candidates across the 47 cohort cases with reported molecular diagnoses of exome variants in OMIM Morbidmap genes. Our tool outperformed Phen-Gen, eXtasy, PhenIX, PHIVE, and hiPHIVE in the prioritization of these clinically reported variants.

**Conclusions:**

Our integrative paradigm can improve efficiency and, potentially, the quality of genomic medicine by more effectively utilizing available phenotype information, catalog data, and genomic knowledge.

**Electronic supplementary material:**

The online version of this article (doi:10.1186/s13073-016-0261-8) contains supplementary material, which is available to authorized users.

## Background

**Fig. 1:**
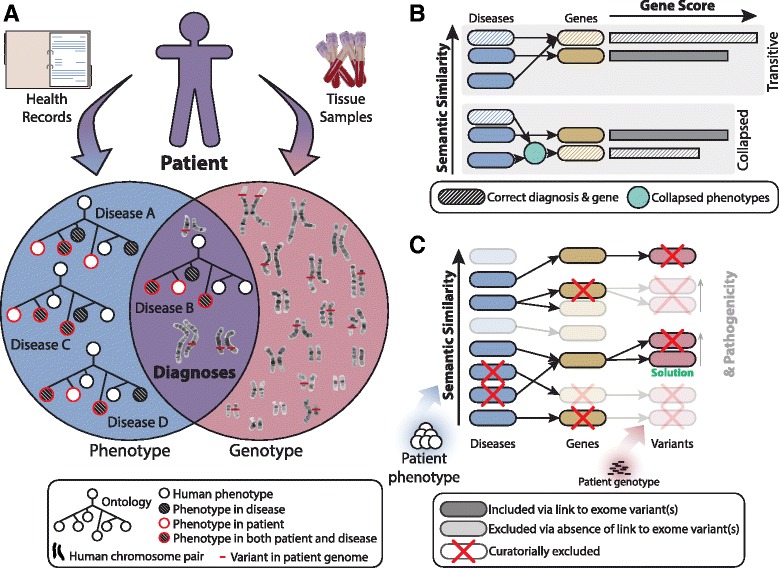
Integration of phenotype with genotype in clinical diagnostics of genetic disease. **a** The diagnostic process is informed by both phenotype and genotype data to arrive at diagnostic conclusions. During the clinical evaluation of patients with suspected genetic disease, physicians observe phenotypic features, and these can be represented in controlled vocabularies (e.g., Human Phenotype Ontology, HPO), amenable to subsequent computational analysis. Physicians also request the acquisition of blood or other tissue samples for molecular characterization of the patient via genome-wide analyses, such as next-generation sequencing. Genotypic analysis provides high-resolution information concerning the location, type, and zygosity of variants within the patient genome. Integration of these data identifies possible solutions that simultaneously match both phenotype and genotype of the patient, excluding unlikely diagnostic candidates and improving differential diagnosis. **b** Our transitive prioritization approach ranks genes and the variants they harbor against patient phenotype as a function of the discrete disease scores with which the genes were previously associated. This avoids potential underweighting and corresponding ranking inaccuracies resulting from the collapsing approach or direct term-to-gene HPO annotations. **c** We implemented a curatorial and visual transitive closure approach to infer phenotypic prioritization scores for patient genotype variants. These scores are based on clinical indication similarity scores computed for diseases in the catalog that are reportedly caused by variants in genes that contain filtered patient variants. When multiple diseases are cataloged to result from variants in the same gene, we determine the gene’s score by aggregation of the scores of those diseases using an integrative function. Variants then inherit the scores of the genes in which they are located. The manual curatorial exclusion of diseases or genes from consideration for diagnosis transitively propagates to eliminate genes and variants from the differential

Genome-wide technologies, including next-generation sequencing, have become increasingly affordable, rapid, and clinically utilized, particularly in comparison to single gene screening. These revolutionary advances in data acquisition have made large-scale genotyping an essential tool for genetic diagnostics and the identification of novel deleterious variants potentially contributing to disease. They hold great promise for the future of molecular diagnosis and management of patients with genetic disease [1–6]. Such technologies also provide particular opportunity for the identification of causes of rare and orphan diseases, which until recently have suffered from a lack of computational tools to help bridge clinical genomics and medical phenotyping and to facilitate diagnostics [7–10]. Despite the promise of available data, the scale of variation presents an interpretive challenge: an individual patient’s genome can have hundreds of rare and putatively deleterious candidate causal variants [11]. Although in some instances diagnostic conclusions can be made without extensive interpretation (e.g., aneuploidies or nonsense variants in disease genes), the presence of numerous potentially deleterious variants typically requires substantial curation to identify the candidate deleterious variant(s) that best matches the clinical phenotypes of the patient in question [1–6, 12, 13]. The goal of integrated diagnostic approaches is to bring together variant knowledge with clinically ascertained patient phenotype characteristics to reach the best-informed diagnostic conclusions (Fig. 1a).

Coincident with the rise of genome-wide data for diagnostics has been the development of standards and catalogs for clinical sign-out [14–16]. Much focus has addressed distinguishing clearly deleterious variants from other variants with less clear contribution to disease. Central to these efforts has been the development of compendia for matching observed variation to well-vetted disease information [11, 17]. Some variants cataloged as “deleterious” can also appear in unaffected individuals, and therefore additional tools have become necessary to identify from among the many candidate variants in affected individuals the specific variants or variant combinations—such as variant pairs for recessive disease—that may explain observed phenotypes [18].

Parallel to the development of catalogs and standards for variant analysis has been the development of systematic tools for representing patient information. The Human Phenotype Ontology (HPO), initially constructed in 2008, is a representation of the features of human disease and the hierarchical relationships that exist among them [19]. A key application of this work is The Phenomizer, a software tool for making comparisons of known diseases to patient phenotypes [20]. This tool uses semantic similarity methods to match patient characteristics, as represented in the HPO, to the Online Mendelian Inheritance in Man (OMIM) disease catalog, which is also mapped to the ontology. The Phenomizer returns candidates within the differential diagnosis as lists and tables, with scores representing the quality of the match [1–6, 20].

The goal of variant prioritization is to construct an ordered ranking of observed genetic variation. This objective differs from that of a differential diagnosis, the fundamental purpose of the Phenomizer. To bridge the gap between disease rankings and gene or variant rankings, extensions of this initial approach have been developed and applied to genome-wide diagnostic data. Two such tools are PhenIX [11, 18, 21] and Phenomantics [21], which directly leverage the Phenomizer’s semantic similarity calculation to consider genome-wide genotypic data. Both PhenIX and Phenomantics match query phenotypes to genes by collapsing phenotypes across the diseases to which a gene’s variants have been associated. This approach therefore effectively considers hybrid diseases for use in semantic similarity calculations. Such collapsing may be problematic because it can result in both overestimation and underestimation of semantic similarity matches of candidate genes to patient characteristics (Fig. 1b). Furthermore, these disease diagnostic intermediates are embedded within the computational scheme and hidden from the user, preventing user-informed exclusion of ruled-out diseases from diagnostic consideration.

Phen-Gen [22] is an alternative approach that employs a Bayesian framework to integrate semantic similarity calculation with proteomic and variant pathogenicity data. Although this procedure retains diagnostic intermediates and does not collapse phenotypes across diseases causally linked to variant genes, it still does not permit additional data input to update or redirect analysis based on initial results. In addition, this tool is more computationally intricate than PhenIX because it recruits protein–protein interaction (PPI) data into its analytic process. By including the protein interaction neighborhood in the variant analysis, Phen-Gen relaxes the distinction between matching a catalog of known causal variant genes and the more exploratory process of disease gene discovery. PHIVE is another algorithm that combines variant pathogenicity scores and catalogs with phenotype similarity analysis using human and mouse data to rank variants, while hiPHIVE uses human, mouse, and other model organism data to do so [23, 24]. Alternatively, eXtasy ranks variants by combining input phenotype similarity scores with scores computed between input genotype data and “fused” human and non-human genomic data, whereas Phevor combines input phenotype data with data from human and non-human ontologies to reprioritize externally pre-computed ranks [25, 26]. As with Phen-Gen, the inclusion in these analyses of gene-to-phenotype data from non-human sources may blur the line between disease gene discovery and clinical application. Other tools, such as PhenoDB [12, 13, 27] and PhenoTips [14, 28], facilitate the collection, classification, analysis, and sharing of clinical indication data, but they do not provide a phenotypically supported connection to particular variants detected in individual patients.

Another challenge for computational tools is the interactive integration of diagnostic or biomedical expertise into the variant analysis process. Aside from brief initial configuration settings, most available tools execute variant prioritization in a single step starting from initial user input. Such approaches limit users from exercising medical judgment to constrain, update, or curate algorithmically determined initial results [17, 18, 21].

We hypothesized that molecular diagnostics could be improved through the application of a transitive prioritization scheme that links phenotypes to variants through medically recognized disease intermediates (Fig. 1c). Moreover, we hypothesized that by coupling this prioritization to a visual and interactive user interface, we could better recruit users’ expertise to improve the diagnostic process beyond that of methods driven by computational algorithms alone. To pursue this approach, we developed novel web-based software employing methods from statistical visualization, software engineering, and semantic similarity analysis. We assessed our tool using the existing OMIM catalog mapped to the HPO [19]. We examined the ability of our scheme to recover known substructures in this catalog—in particular, its ability to distinguish disease classes as previously defined by the Human Disease Network (HDN) [29], as well as the OMIM Phenotypic Series [18, 29, 30]. We then applied our method to exome variant data previously analyzed by the Baylor Miraca Genetics Laboratory (BMGL) [31]. Our work demonstrates that the visual interactive approach is practical and produces results that closely match those of expert review, while simultaneously extending the framework of semantic-similarity-based analysis. We also elaborated this tool with a clearly separated function for variant discovery driven by semantic similarity methods. Collectively, these advances represent important contributions in the area of algorithms and software development for genome-wide variant analysis.

## Methods

### Semantic similarity

Semantic similarity is a computational technique that compares sets of terms within a domain of knowledge. The technique relies on controlled vocabularies, such as ontologies, to compute approximate matches between queries and related vocabulary terms [32]. In the diagnostic context of human clinical phenotype analysis, semantic similarity calculations quantitatively compare patient phenotype term sets to sets defined by a catalog of known diseases or syndromes. We used as the substrate for our calculations the HPO mapping of the OMIM catalog, which provides descriptions of thousands of known genetic diseases and the corresponding genes in which causative variants have been observed [20, 30, 33–35].

A variety of semantic scoring methods have been developed. These scoring methods can be broadly grouped into two primary categories: (a) scoring approaches that use the ontological topology alone and (b) approaches which explicitly depend on catalog annotations to the ontology. Topology-only scores focus exclusively on the relationship structures between terms within an ontology (e.g., the HPO) [36]. Similarities are determined by traversing the directed acyclic graph to compute characteristics of shared ancestry and descendants between collections of nodes comprising queries and the target database. One such method is the GO-Universal method that functions by determining the “topological reachability” of each ontological term. Distinctly, annotation-based methods compute scores based on catalog annotations to an ontology. Of particular importance for these annotation-based scores is the concept of information content—a logarithmic transformation of rareness of annotations at or below each term as determined by association of the knowledge catalogs (e.g., the set of OMIM diseases) to the ontology.

To compute annotation-based similarities, we used a version of the Resnik method [37], as symmetrized by Köhler, *et al.* [20]. In what follows, let *D* = an annotated disease, *Q* = a queried phenotype term set, *d*{*t*} = set of diseases annotated with term *t*, *A*{*t*} = set of terms *t* and all their respective ancestors, *C*{*t*} = set of terms *t* and all their respective children, and *||x||* = quantity of elements in set *x*. Let *N* be the total number of disease in the catalog that are annotated to the ontology. The symmetrized Resnik calculation is defined:

$$ {S}_R\left(D,Q\right)=\frac{1}{2}\left(avg\left[\sum_{t_1\in D}\underset{t_2\in Q}{max}\left[\underset{t_j\in A\left\{{t}_1\right\}{\displaystyle \cap }A\left\{{t}_2\right\}}{max}\left[ log\kern0.28em \left(\frac{\parallel d\left\{C\left\{{t}_j\right\}\right\}\parallel }{N}\right)\right]\right]\right]\right)+\frac{1}{2}\left(avg\left[\sum_{t_1\in Q}\underset{t_2\in D}{max}\left[\underset{t_j\in A\left\{{t}_1\right\}{\displaystyle \cap }A\left\{{t}_2\right\}}{max}\left[ log\kern0.28em \left(\frac{\parallel d\left\{C\left\{{t}_j\right\}\right\}\parallel }{N}\right)\right]\right]\right]\right) $$

We also implemented an ancestral term overlap (ATO) method for computing semantic similarity. This method sums the unique overlap between pairs of phenotype sets, including their ontological ancestry. The ATO differs from the previously reported term overlap method [38] in that all ontological nodes shared between a pair of phenotype sets are included in the calculation:

$$ {S}_O\left(D,Q\right)=\kern2mm \parallel A\left\{{t}_i\in D\right\}\cap A\left\{{t}_j\in Q\right\}\parallel $$

In an effort to optimize resolution of differences among scored diseases, we examined weighting schemes to extend the ATO by using catalog information content [37] and weights determined by the topological information specified for the GO-Universal method [39]. We used the R statistical programming language to implement our calculations [40]. Because annotation to a knowledge catalog is required for calculation of the catalog-based information content, we excluded from catalog-weighted similarity analysis all HPO terms for which there exist no annotations to the OMIM catalog. Conversely, owing to the nonlinearly decaying nature of the GO-Universal calculation, a “reachability” topological position characteristic *TPC* of 0 was computed for 322 low-depth HPO terms, resulting in an infinite topological information content *TIC =* −log(*TPC*). We compensated for this by manually assigning to these terms a *TIC* of 2.225074 × 10^−308^, the machine minimum for the R language.

### Semantic similarity analysis of known disease classes

We analyzed known collections of similar disease classes previously and independently defined as disease classes by the OMIM Phenotypic Series and the HDN [29, 30, 41]. Hypothesizing that diseases should be highly similar within classes, but distinguishable between classes, we used Resnik semantic similarity to calculate average scores between disease pairs within the same classes and compared these scores to those between pairs across different classes. For each class, we computed as a signal-to-noise ratio the quotient of mean within-class similarity and mean between-class similarity.

### Input data

The phenotypic component of the input, or query, to our analysis is a set of HPO terms describing the clinical presentation of a patient. The genotypic component is a set of genes or gene variants. This genotype may be provided as a simple gene list or in the form of a variant call file (VCF), typically generated as a summary of next-generation sequencing results. The provided list is expected to be filtered to remove common variants (e.g., >1 % population minor allele frequency [MAF]) or restricted to variant classes known to be inactivating mutations (e.g., frameshift or nonsense). Although our software is informed by the ExAC database (v0.3) [42] to annotate variants with observed frequencies, our software is not currently intended to perform this variant-frequency-based filtering step, but expects this processed content as input.

### Natural language processing of free text for phenotypes

To facilitate construction of query phenotype sets from raw clinical notes, we used the Bio-Lark Concept Recognizer application programming interface to provide natural language processing for automated extraction of HPO terms from input clinical presentation text narratives [43]. We enabled automated export of these results in our software to use these extracted phenotypes in subsequent semantic similarity analysis.

### Query-based disease prioritization

We used semantic similarity and HPO annotations to estimate scores describing similarities of an input query to the 7,746 OMIM diseases defined in terms of the HPO phenotypes [19, 30, 44]. As described above, the phenotypic input to our analysis is a set of HPO characteristics, such as those observed during clinical examination of a patient or provided as indications for testing. To calculate diagnostic rankings of disease, we compute similarity scores via Resnik, ATO, ATO weighted by the GO-Universal information content, or ATO weighted by annotation-based information content algorithms. For each query, scores are computed for 7,746 diseases. We optionally limit the ranked disease list to diseases that also have OMIM Morbidmap [30] annotations, are restricted to particular genetic models (e.g., have only dominant or recessive inheritance), contain user-defined required phenotypes, or are causally linked in OMIM Morbidmap to genes identified as having candidate variations in the patient.

### Transitive prioritization of variants

We use a transitive closure approach to infer scores for the input variant gene set based on scores matching phenotype queries to disease. The scores are restricted to diseases in the catalog that are mapped by OMIM to genes harboring variants in the input set. For all diseases *d{G}* cataloged to result from variants in a gene *G*, we use an integrative function *F* to determine the transitive diagnostic relevance score *S*_*T*_ for *G* against phenotype query *Q* by aggregating the *d{G}* similarity scores:

$$ {S}_T\left(G,Q\right)=F\left(\underset{D_i\in d\left\{G\right\}}{\mathrm{S}}\left({D}_i,\mathrm{Q}\right)\right) $$

We tested the mean, maximum, and sum as aggregation alternatives for *F*. To permit comparison between the transitive prioritization approach and alternatives, we implemented the direct gene scoring approach used by Phenomantics [21], which analyzes the HPO *term-to-gene* annotations, and that used by PhenIX [18], which analyzes the unions of phenotypes collapsed from all diseases associated with each gene via the OMIM Morbidmap [30].

### Genetic models

Our software implements an optional feature to impose constraints determined by models of inheritance of genetic disease. This feature rules out differential intermediate diseases whose variant attributes do not meet inheritance requirements. For autosomal dominant disease, a single heterozygous variation is sufficient to cause disease; when recessive disease is suspected, both copies of an autosomal gene must be impacted for disease to result. Invoking the logic of the recessive model, the software restricts differential matching consideration to diseases causally linked to genes with homozygous variation or where compound heterozygous variation is possible based on the presence of two or more qualified variants within a gene. Once imposed, the inheritance model dictates disease filtering that transitively propagates to variant prioritization in the tool. The default mode of our software imposes no constraint for suspected model of inheritance.

### Global visualization

To create a global visualization of all 7,746 phenotype-annotated diseases in the OMIM catalog, approximating their similarities to each other and to individual patients, we applied classical multidimensional scaling (MDS) to semantic similarity calculations. MDS is a well-established statistical procedure that has been extensively documented [45] and requires semantic-similarity-derived dissimilarities as input. To transform similarity to dissimilarity, we subtracted each score from the maximum observed score, so that the maximum similarity between a pair of diseases has a corresponding dissimilarity of 0 and the minimum similarity has the largest dissimilarity. MDS determines a low-dimensional projection as output. This procedure renders the *n* × *n* dissimilarity matrix into an *n* × *k* matrix, and for *k* ≤ 3 can be visualized as a low-dimensional best-fit map of OMIM diseases [45].

To contextualize a patient on this map, we calculated a convex combination of coordinates as the similarity-weighted location determined by nearest *m* semantic neighbors (e.g., the top five diseases most similar to the query). To make the weights sum to one, weights of each neighbor are determined by dividing each disease similarity by the sum of similarities of the *k-*nearest neighbors. The choice of *m* is a user-defined parameter, defaulted at 5.

### Local visualization: radar plot

To create a local visualization of only the top *n* semantic disease matches to a phenotype query, we constructed an alternate two-dimensional visual display. This local map utilizes distance from the center to strictly represent diseases according to their exact similarities to the query. We place the query itself at the origin and linearly transform disease similarity scores into dissimilarity distances via the equation below. In what follows, the radius *r*_*D*_ of disease *D* is calculated as a function of the similarity *S* of a query *Q* to itself and to *D*.

$$ {r}_D=\frac{S\left(Q,Q\right)-S\left(Q,D\right)}{S\left(Q,Q\right)} $$

The circumferential placement of diseases is determined by a one-dimensional MDS analysis of the *n* candidate diseases and represents the best one-dimensional approximation of the similarities of the *n* candidates to each other. To circumferentially spread the *n* candidates according to their similarities to each other, we scale the observed range of MDS across 360 degrees. To overlay attribute data for input variants in genes causally linked to the *n* candidates by the OMIM Morbidmap [30], we logarithmically scale candidate point size by variant frequency in the ExAC database [42] and linearly scale candidate point color by variant pathogenicity score computed by MutationTaster [46]. We manually assign a pathogenicity score of 1 to all exonic frameshift variants for which MutationTaster scores are not returned. Owing to its appearance, we refer to this local two-dimensional representation as a “radar plot.”

### Diagnostic curation

The identification of differential intermediate disease rankings in our transitive prioritization approach presents a unique opportunity for clinicians to interact with and curate results through our visual tool. Via the toggle interface embedded into the radar map of our interactive software, users can click diseases to “exclude” from the differential the candidates that they are able to rule out. Subsequently, the variant-associated ranking of diseases excluded or “ruled out” from diagnostic consideration are not included in the calculation of gene-level scores, directly modifying the transitive prioritization of variants.

To enhance this curatorial process, we implement a “hovered disease” functionality to provide an instantaneous, detailed display of input variants in genes associated with the hovered disease as well as available MAF and variant pathogenicity data. The hover function also presents for the disease the complete set of known HPO phenotype associations, that is, the subset of phenotypes shared between the disease and query, incorporating ontological ancestry to perform approximate matches between phenotypes.

### Phenotype suggestion

Analysis of phenotype and genotype queries can narrow the differential to a subset of disease candidates that are distinguished by particular phenotypic characteristics. We implemented a procedure to propose that such diagnostically informative phenotypes be considered for addition to the query. For each phenotype query, we calculate these suggestion characteristics as the rarest non-query phenotypes annotated to the diseases most similar to the query.

### Analysis of exome data

We evaluated the performance of our transitive prioritization approach on the exome variants reported for a previously published cohort of genetic disease patients [31]. We obtained the detailed data from the Whole Genome Laboratory at BCM, now BMGL. Patient phenotype information was encoded into the HPO by manual review of input clinical forms for 245 cases. Filtered variant gene sets were obtained for 49 (96.1 %) of the 51 cases with reported diagnoses and 158 (81.4 %) of the 194 cases without reported diagnoses. Allele-specific variant details were obtained from Exome VCF files for 47 (92.2 %) of the 51 cases with reported diagnoses and 157 (81.0 %) of the 194 cases without reported diagnoses. For each of these cases, we integrated the encoded phenotype data with the VCF data to compute transitive prioritization ranks for the reported variant gene(s). We limited our transitive evaluation to the 47 solved cohort cases with (1) reported molecular diagnoses of variants in OMIM Morbidmap [30] genes, (2) exome data available in VCF files (median quantity of variant genes = 464), and (3) signed-out variant cataloged in ClinVar [17]. For analysis by our program, “OMIM Explorer” (OE), gene symbols were extracted from VCF data and ranked via transitive prioritization; for HPO-direct and Morbidmap-collapse analysis, these gene symbols were ranked directly via Resnik semantic similarity; and for comparator tool analysis, case phenotype and VCF data were provided to Phen-Gen [22], eXtasy [25], PhenIX [18], PHIVE [23], and hiPHIVE [24] to rank variants. To convert gene aliases into approved HUGO Gene Nomenclature Committee gene symbols for comparator analysis, we used the org.Hs.eg.db package (November 2015 release) for the R statistical programming language. This step was accomplished by mapping each gene symbol to its Entrez Gene identifier and then mapping the Entrez identifier back to the corresponding official gene symbol. This approach was used to check and remap gene symbols as reported by the BMGL as well as those annotated by the comparator tools.

### Novel gene and variant discovery

Patients may present with variants in genes that are not cataloged as previously known to cause disease. We developed an algorithm for semantically driven disease gene discovery to provide a facility for discovering new gene-to-disease associations, an operation distinct from catalog-based variant prioritization. First, we transitively use patient phenotype-to-OMIM similarity scores to identify the set of genes mapped to diseases most similar to the patient phenotypes. We then use an external knowledge source—in our case, the PINA 2.0 PPI network [47]—to identify candidate genes as those genes that are variant in the patient and highly connected to the training genes. We explored a variety of scores to rank candidates, including quantity of connections to training genes and percentage of total connections of a candidate that are training genes. The latter determines the default ordering of gene results in our tool.

### Variant reference data

Variant frequency data were obtained from the ExAC Exome Aggregation Consortium (ExAC, v0.3), Cambridge, MA, USA [42]. Variant pathogenicity data was computed by MutationTaster and accessed via the Bioconductor package rfPred [46, 48].

### Webtool

We used RStudio Shiny [49], a web application framework for the R statistical programming language, to create an interactive, stateful implementation of our transitive variant prioritization and disease gene discovery workflow. We have named this novel software “OMIM Explorer” (OE) and made it available at http://www.omimexplorer.com. Links at the site also provide access to detailed tutorial videos describing the intended use of software features and providing step-by-step instructions.

### Session statefulness

The state of an OE session includes the visualization settings, discovery settings, phenotype sets, variant sets, free text clinical summary content, and user-supplied curation to exclude specific disease from the differential. The state of an OE session determines the ranking of diseases, variants, and disease gene discovery candidates via the semantic-similarity-based transitive closure logic. Changes in session state immediately propagate to changes in the ranking of diseases and variants. Users can save, download, and share OE session files. These files can also be archived for future use.

## Results

### Semantic similarity analysis of known disease classes

To assess the performance of semantic similarity, we conducted analyses using known classes of related disease defined by the OMIM Phenotypic Series and the HDN classes. We restricted analysis to Phenotypic Series groups comprising six or more disease entries that were annotated to the HPO, ensuring meaningful comparisons [30]. We performed within-versus-between calculations for these disease classes. We found that within-class similarities were substantially higher than those between classes: signal-to-noise ratios were consistently well above one, indicating strong signal in the semantic scores. The mean similarities between classes were consistently low and uncorrelated with class composition. We observed similar tendencies with disease groups defined by the HDN [29] (Additional file 1: Figure S1A, B).

### Visualization of disease catalogs and differential diagnosis via semantic similarity

**Fig. 2:**
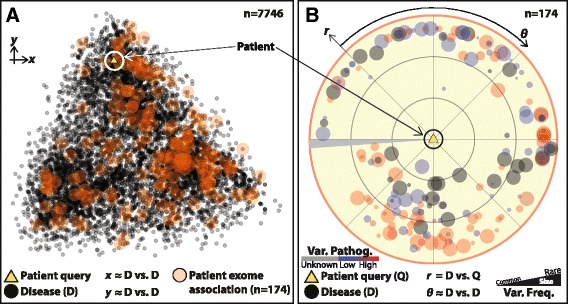
Visual representations of differential disease spectra. In this case selected from a retrospective genetic disease cohort, bradykinesia, developmental regression, dystonia, motor delay, delayed speech and language development, and ptosis were reported. **a** The global map: a visual map representation of the relationships between the phenotypic features of the case (*yellow triangle*) and all 7,746 cataloged Online Mendelian Inheritance in Man (OMIM) diseases (*gray circles*). The two-dimensional space *x*,*y* is defined by the first two multidimensional scaling (MDS) components computed from Resnik similarities between all pairs of diseases. A projected map location is calculated for the case via a weighted convex combination of the coordinates of the diseases with the top five similarities to the complete patient phenotypes. The genotypic spectrum of disease for the case, comprising the 174 diseases with known causes in genes variant in the filtered patient exome, is highlighted in *orange* and is clustered throughout the visual space. **b** The local/radar map: an improved visual map representation of the relationships between the phenotypic features of the selected clinical case and the 174 genotypic spectrum diseases. The case is placed at the center of the map. The circumferential disease distribution across 360 degrees *θ* is a linear scaling of the first MDS component computed from Resnik similarities between all pairs of diseases. Radial dissimilarity distance *r* is computed for each disease as the linear transformation of its similarity to the patient phenotypes. The size and color of disease points indicate the ExAC [42] and MutationTaster [46] pathogenicity of case variants harbored in genes causally linked to top differential intermediate diseases. The map can be progressively filtered to reflect mandatory aspects of clinical phenotype and genotype or manual curation of differential intermediates, as performed by clinicians

The differential of potential disease diagnoses is essential to the logic of transitive prioritization. We hypothesized that visual engagement with these diseases would clarify their role and help improve molecular diagnostics and disease gene discovery. We used MDS of our high-dimensional similarity calculations in semantic space to generate a low-dimensional projection—a global map in visual space—of the 7,746 diseases in the OMIM catalog annotated with HPO phenotypes, making inter-disease relationships easier to conceptualize (Fig. 2a). The resulting approximate visualization of the relationships between all pairs of diseases and between each disease and the case successfully maintained the within-group relationships for known HDN and OMIM Phenotypic Series disease classes in semantic similarity space (Additional file 2: Figure S2). Additionally, we observed in three-dimensional visual space the colocation of strictly-defined Phenotypic Series classes (e.g., specific eye and skeletal diseases) within their more broadly defined HDN class counterparts (e.g., all eye and skeletal diseases, respectively) (Additional file 3: Figure S3).

We projected into this map a case from our published exome cohort [31], in which bradykinesia, developmental regression, dystonia, motor delay, delayed speech and language development, and ptosis were reported. We computed coordinates for the case location as the convex combination of the coordinates of the five diseases most similar to the reported phenotypes. We then identified an ordered differential of diseases potentially causing the observed phenotypes in the case by highlighting all 174 diseases linked to filtered exome variant data. We linked the diseases to variants through OMIM Morbidmap [30] indications that suggested the 174 diseases were previously observed to be caused by variants in genes that also contained potentially pathogenic rare variant alleles in the personal genome of the patient (Fig. 2a). Overall, global map projections were relatively accurate (Additional file 4: Figure S4A), and allowed for simultaneous representation of the proband, together with all 7,746 annotated diseases. We observed, however, that diseases very similar to the reported phenotypes in semantic space were neither consistently nor sufficiently close in visual space, and vice versa (Additional file 4: Figure S4B). In conjunction with our convex combination coordinate projection, the MDS-inherent mathematical compromises responsible for these inadequacies therefore yielded maps that were too inaccurate for inferring exact diagnoses from visual relationships between a projected patient and differentials.

To remedy the limitations of the global map, we developed the local “radar map” alternative display. This plot places the top differential intermediate diseases at semantically accurate dissimilarity distances from the phenotype input for a case (Fig. 2b). It also presents the approximate semantic similarity relationships among candidates as determined by one-dimensional MDS, which is represented in the circumferential spacing of points. The one-dimensional MDS retains relatively accurate approximations of the relationships that exist among the diseases. Furthermore, rather than highlighting a subset of the entire catalog corresponding to the input genotypic and phenotypic spectra of diseases, the radar map progressively filters its contents to these spectra as defined by the user and modifies both the size and color of disease points to represent disease similarity to the patient, the MAF and pathogenicity of input variants in causally associated genes, and manual curation of differential intermediate diseases performed by clinicians.

### Application to exome data

**Fig. 3:**
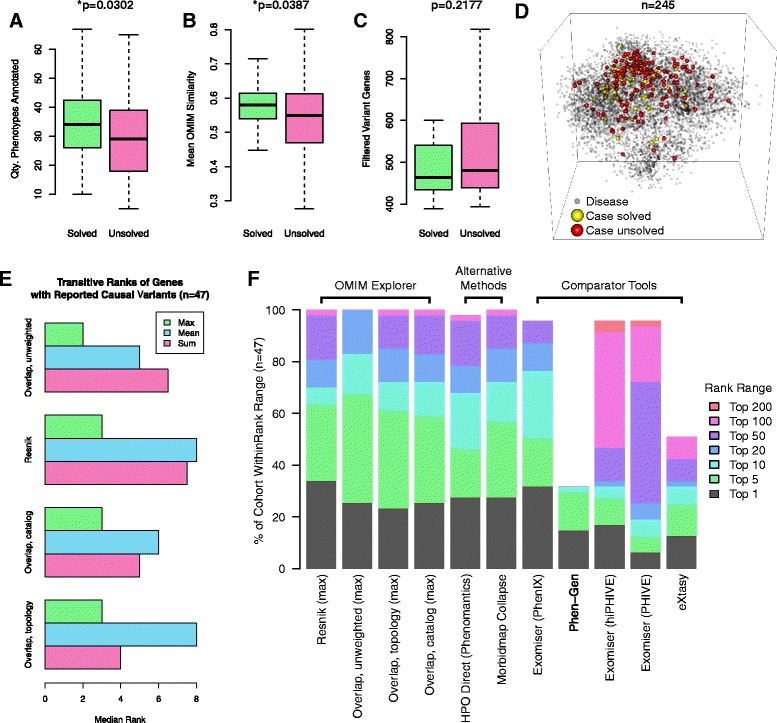
Solved and unsolved cases in the BMGL cohort. In 245 exome cases, 51 had reported molecular diagnoses. The solved cases tended to have (**a**) more Human Phenotype Ontology (*HPO*) phenotypes, including ontological parent terms (Wilcoxon *p* = 0.0302); **b** higher average similarity to the Online Mendelian Inheritance in Man (*OMIM*) catalog (Resnik similarity, Wilcoxon *p* = 0.0387); and (**c**) lower quantities of filtered variant (Wilcoxon *p* = 0.2177). **d** Visualization. Multi-dimensional scaling representation of the 51 solved (*yellow spheres*) and 194 unsolved (*red spheres*) cohort cases in a three-dimensional map of all 7,746 cataloged OMIM diseases (*gray spheres*). Solved and unsolved cases appear similarly distributed in the visual space. **e** Transitive method comparison. Across the 47 solved cases with reported Morbidmap genes, we tested maximum, mean, and sum as aggregation function alternatives; semantic similarity was calculated using symmetrized Resnik, unweighted ancestral overlap, and versions of ancestral overlap weighted by OMIM catalog information content and the topological information specified for the GO-Universal method [39]. Globally, the transitive maximum achieved the lowest median rank. **f** Comparison of relative performance. Phenotype and filtered genotype data for 47 cohort cases with reported molecular diagnoses were analyzed via the transitive maximum OMIM Explorer algorithms, phenotype-collapsing alternative algorithms, and comparator tools. A minor allele frequency (MAF) filter of 1 % MAF was applied in PhenIX, PHIVE, and hiPHIVE. Because eXtasy limits the quantity of phenotypic inputs to 10, we supplied eXtasy with only up to the 10 phenotypes with the highest information content (i.e., rareness in the OMIM catalog) scores for each case. Via OMIM Explorer, transitive maximum aggregation (Resnik) returned a top ranking for 16/47 = 34.04 % of the cohort and a ranking in the top five for 30/47 = 63.83 %; the overall best alternative, PhenIX, returned a top ranking for 15/47 = 31.91 % and a ranking in the top five for 24/47 = 51.06 %

Of the 245 genetic disease cases in a retrospective cohort of individuals referred for whole exome sequencing, we analyzed the 51 for which a molecular diagnosis was reported [31]. The molecularly diagnosed cases tended to have more phenotypes and higher similarities to the OMIM disease catalog, while those undiagnosed tended to have higher quantities of variant genes after frequency and synonymy filtering (Fig. 3a–c). However, both classes of case were equally distributed in the visual space of the global disease map (Fig. 3d). For 47 of these 51 cases, the reported variant genes were associated with diseases via the OMIM Morbidmap [30]. With the assistance of the Bio-Lark Concept Recognizer [43], we manually reviewed the clinical notes for these 47 solved cases and updated their phenotype annotations to a more recent instance of the HPO. We used these updated annotations to compute cumulative distribution curves to evaluate the performance of OE across each of the 47 solved cases (Fig. 3f). We employed our transitive maximum as the integrative aggregation function because the maximum, rather than mean or sum, associated disease similarity score determined gene ranks that best matched those generated by the diagnostic laboratory (Fig. 3e). We observed that our transitive maximum prioritization approach implemented in OE computed median ranks of 2 via the term overlap method and a median of 3 via symmetrized Resnik similarities for the previously reported variants in these cases (Fig. 3f). Given that the median quantity of filtered variant genes identified in each of the 47 cases was 464 (Fig. 3c), the transitive maximum overlap and Resnik similarity approaches assigned to the reported variants median ranks in the top 1 % of all filtered variants (Fig. 3f).

We compared the performance of the OE transitive maximum to that of Phen-Gen [22], eXtasy [25], PhenIX [18], PHIVE [23], and hiPHIVE [24]. Because the latter three were implemented via Exomiser, which allows for variant filtration by MAF, we applied a filter of 1 % MAF. Because eXtasy limits the quantity of phenotypic inputs to 10, we input the phenotypes with the top 10 information content scores to eXtasy for each of the 47 cases analyzed. We observed that for our cohort, OE returned scores for reported variant genes for more cases and had lower median ranks for the reported genes than did four of the five comparator tools. Phen-Gen failed to return scores for the clinically reported gene variants in 32 of the 47 cases (68.09 %); however, when Phen-Gen returned scoring results for the reported gene variants, it performed the best among all tools, with a median rank of 1.5 across these 15 of 47 cases (31.91 %). We also observed that the OE Resnik transitive maximum algorithm outperformed the best-overall-performing comparator tool, PhenIX, which yielded a median rank of 5 for the reported results on the test cases. OE returned a top ranking for the causative variant in 16 of the 47 cases (34.04 %) and a ranking in the top five for 30 out of 47 cases (63.83 %), while PhenIX returned a top ranking for 15 out of 47 (31.91 %) and a ranking in the top five for 24 out of 47 (51.06 %; Additional file 5: Figure S5 and Additional file 6: Table S1).

### Case study

The radar plot implements curatorial interactivity using semantic similarity to identify candidate diagnoses. This plot presents accurate semantic similarity relationships of cases to differential disease candidates and visually distributes them according to their pairwise relationships. The web-based interactivity of this plot provides heads-up display information identifying each candidate, describing its phenotypic match to the query and distinction from alternate candidates, and presents corresponding variant information. To examine the plot’s performance in detail, we analyzed a single solved case from the retrospective cohort [31]. The patient in that case exhibited phenotypes of sinus bradycardia, pericardial effusion, delayed central nervous system myelination, epileptic encephalopathy, gastroesophageal reflux, encephalopathy, microcephaly, intellectual disability, and seizures. Whole exome sequencing of DNA extracted from whole blood led to the identification of 928 candidate variants in 837 genes, after filtering for variant frequency and changes to protein coding. Of these genes, 145 were cataloged in the OMIM Morbidmap [30] to harbor disease-causing variants. The BCM diagnostic laboratory reported as potentially causal a nonsynonymous variant detected in the *SCN8A* gene, in which defects cause early infantile epileptic encephalopathy (MIM #614558) and cognitive impairment with or without cerebellar ataxia (MIM #614306) [31].

**Fig. 4:**
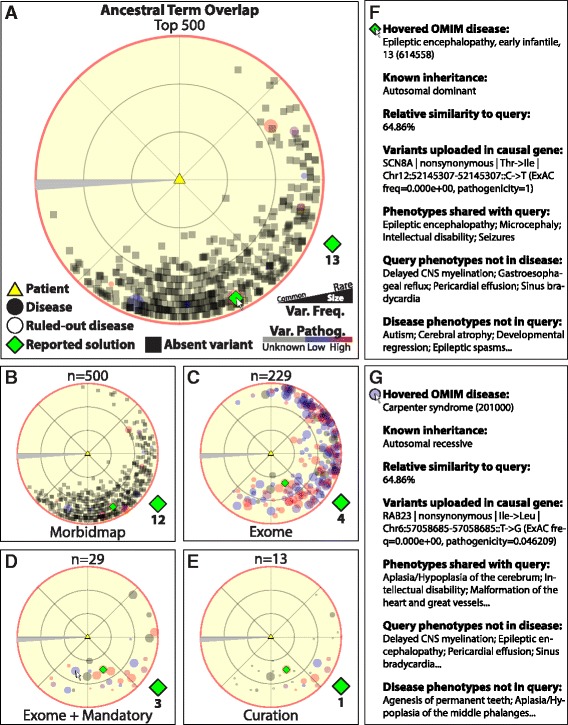
OMIM Explorer radar map performance on a solved clinical case study (unweighted overlap similarity). The patient in the case (*yellow triangle*) had indications of sinus bradycardia, pericardial effusion, delayed central nervous system (*CNS*) myelination, epileptic encephalopathy, gastroesophageal reflux, encephalopathy, microcephaly, intellectual disability, and seizures. The filtered exome identified candidate variation in 145 Online Mendelian Inheritance in Man (OMIM) Morbidmap genes. Variants were ranked via transitive maximum unweighted ancestral term overlap similarity. **a** Top candidate diseases (TCDs) of the differential intermediate. The 500 TCDs by semantic similarity (*colored circles*) are represented in the radar map. The reported *SCN8A* variant [ClinVar: SCV000245399.1] present in the patient is transitively ranked at 4 via the MIM #614558 rank of 13. **b** TCDs with cataloged causal variants. The 500 TCDs are filtered to those with causal gene variants cataloged in the OMIM Morbidmap. The *SCN8A* variant is transitively ranked at 4 via the MIM #614558 rank of 12. **c** Exome-linked TCDs. The Morbidmap TCDs are filtered to 229 diseases associated with genes variant in the patient. The *SCN8A* variant is transitively ranked 4 via the MIM #614558 rank of 4. **d** Exome TCDs with mandatory phenotypes. The 229 exome TCDs are filtered to 29 known to present with intellectual disability as observed in the patient. The *SCN8A* variant is transitively ranked 3 via the MIM #614558 rank of 3. **e** Interactive curation of exome TCDs. Medical knowledge is used to rule out 16 of the 29 remaining TCDs from the differential owing to the absence of their hallmark features. This improved the transitive rank of the *SCN8A* variant from 3 to 1. **f** Display of the variant gene. Early infantile epileptic encephalopathy is caused by variants in *SNC8A*, which is variant in the patient. The detected variant is rare and has high pathogenicity. **g** Display of a curatorially excluded TCD. Carpenter syndrome, caused by variants in *RAB23*, is excluded because characteristic features of skull, hand, or foot abnormalities were not reported

The transitive maximum similarity analysis used the overlap score to automatically assign a rank of 4 to *SCN8A* (Fig. 4a, f) by restricting the differential intermediate to the 229 diseases causally linked via the OMIM Morbidmap to genes variant in the patient exome (Fig. 4b, c). The OE visual curation interface was then used to manually enforce a mandatory phenotype filter, limiting the candidate differential to the 29 patient exome-linked diseases cataloged to present with the intellectual disability observed in the patient (Fig. 4d). Using medical knowledge to guide additional curation, 16 of these 29 diseases were further excluded from the differential intermediate for this case owing to the absence of their hallmark features in the patient, including short stature (microcephalic osteodysplastic primordial dwarfism [MIM #210720], Carpenter syndrome [MIM #201000], Rubinstein-Taybi syndrome [MIM #180849], Wiedemann-Steiner syndrome [MIM #605130]); hand, foot, or nail abnormalities (Carpenter syndrome [MIM #201000], Rubinstein-Taybi syndrome [MIM #180849], Temple-Baraitser syndrome [MIM #611816]); hypoglycemia (hyperinsulinemic hypoglycemia [MIM #256450]); and brain or renal tumors (tuberous sclerosis 2 [MIM #613254]). These interactive curation steps improved the rank of the reported causal variant gene *SCN8A* from 4 to 1 (Fig. 4e, g). A similar performance was observed via a transitive maximum similarity analysis using the Resnik score (Additional file 7: Figure S6).

### Disease gene discovery

**Fig. 5:**
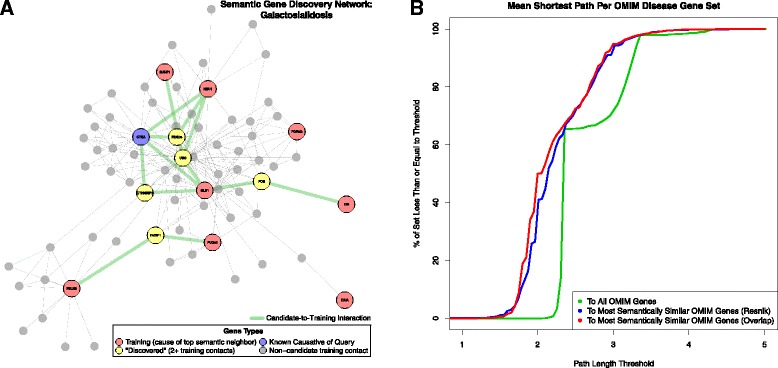
Disease gene discovery via semantic similarity and protein–protein interaction network. **a** Our semantically driven disease gene discovery approach using external omic knowledge. This approach establishes the semantic neighborhood of a patient to identify a relevant known disease gene set, and then recruits prior knowledge of relevant gene–gene relationships to intersect with patient variations. This integration of the disease catalog with omic knowledge results in potential variant discovery and phenotypic extension of known disease genes. As shown in this example, a patient phenotype query determines training genes: those variant in the patient and known to contain variants causing cataloged diseases most similar to the query. The biological subnetwork implicated by these training genes is then realized in “omic space.” For this example, proteomic space, as defined by the PINA2 protein–protein interaction network, is used. This process identifies candidate genes that are variant in the patient and connected to training genes in the protein interaction network. In this figure, an additional constraint has been applied, in which genes must directly interact with at least two training genes to be considered candidates. **b** To validate this procedure we performed a global analysis across the entirety of Online Mendelian Inheritance in Man *(OMIM*)*.* In protein interaction network space, the variant genes of nearest semantic neighbor diseases are typically closer to each other than to those of all diseases

If no adequate diagnostic match is identified via the similarity-driven transitive variant prioritization approach, we provide a novel phenotype–gene association discovery tool that uses the neighborhood of diseases most phenotypically similar to patient phenotypes to determine a phenotypic neighborhood training gene set. We then use prior knowledge in the form of PPI networks to identify candidate genes both variant in the patient and connected in “genomic-annotation space” to the phenotypically implicated training gene set. We used the PINA2 PPI network to perform this analysis [47]. To evaluate the performance of our PPI-based transitive disease gene discovery approach, we applied its algorithm to the HPO representation of OMIM diseases and their corresponding genotypic attributes recorded in the OMIM Morbidmap [35]. We observed that the protein products of genes causative of diseases nearest in semantic similarity space are also closer in PPI space than those of typical disease genes (Kolmogorov–Smirnov *p* < 2.2 × 10^−16^) (Fig. 5a), suggesting the utility of such an approach. A validation example of using the tool to “discover” a gene known to cause human diseases is presented in Fig. 5b.

### Interactive Webtool

We implemented the algorithmic concepts described above into a software system. Our tool also implements the Bio-Lark natural language processing engine [43] to automatically extract HPO terms from clinical narratives for use in semantic similarity calculation, and a wordcloud feature that sizes each input query term according to its relative information content, in comparison to that of other input phenotype terms. Additionally, our tool supports session statefulness, which allows users to save and load their work and share it within collaborative diagnostic teams. Our tool was developed using the RStudio Shiny web application development framework [49]. Shiny uses the R statistical programming environment together with Node.js [50]. This platform allows Shiny, and software developed with it, to take advantage of an event-driven, non-blocking I/O model, which has little computational overhead. OE is an example of a data-intensive, real-time application running across distributed devices. The tool, and links to detailed tutorial videos of example use cases, are publically accessible at http://www.omimexplorer.com.

## Discussion

Genome-wide data interpretation is a central challenge of genomic medicine, and will likely continue to be for years to come. Biomedical software plays a fundamental role in meeting this challenge. We have developed an interactive visual tool to meet this challenge. Our tool is distinctive in several ways. First, our tool allows users to input clinical information as free-text notes that are translated into HPO terms. Second, our analytic approach uses transitive prioritization to rank subject phenotypes against their variant genes using the cataloged associations with known genetic diseases. Third, the tool allows users to update, or fine tune, these ranks using their medical expertise to rule out particular diseases or to impose phenotypic constraints or additional filters. Fourth, curation is driven by a novel visual interface that is both stateful and visually interactive. This visual and interactive approach is iterative, and therefore fundamentally different from previous work that has relied more on single-step computational analysis. Finally, our tool permits the saving of session files for sequential effort, archival, or data sharing.

Although our work may increase the efficiency and effectiveness of human users, it is not a command line tool intended for automated high-throughput use in larger computational pipelines without the interaction of human users (Table 1). While we believe other alternatives in this space are better suited for full automation, such implementations may exclude the contribution of real-time, adaptive medical expertise from the variant prioritization process. Collectively, we believe that our approach better recruits biomedical and clinical experts into the variant analysis workflow, but with the tradeoff that this interactivity requires active input from users.

**Table 1:**
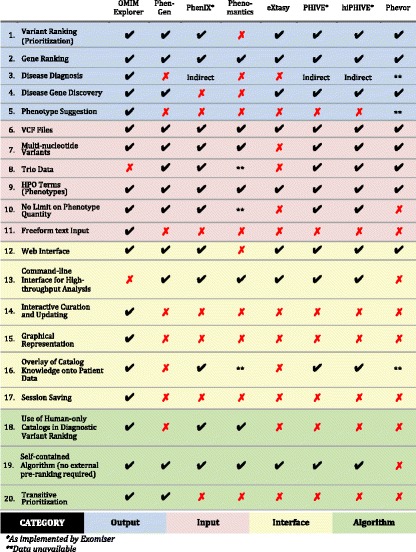
Comparison of tool features

1. Ranking of input variants. 2. Ranking of genes containing input variants. 3. Ranking of diseases. 4. Identification of gene candidates for causal association with input phenotypes. 5. Identification of phenotypes that may help clarify or distinguish among top rankings. 6. Acceptance of variant sets as VCF (variant call format) files. 7. Inclusion of multi-nucleotide (insertion/deletion/frameshift) variants in computational prioritization. 8. Support for integration of family VCFs to distinguish between transmitted and de novo variation. 9. Acceptance of phenotypic query descriptors as HPO (Human Phenotype Ontology) terms. 10. Absence of limit on quantity of input phenotypes (HPO terms) supplied. 11. Acceptance of unstructured text from which input phenotypes are computationally extracted. 12. Accessibility via a web browser. 13. Accessibility via a command line API (application programming interface), which facilitates automated batch submission of distinct case queries. 14. Immediate update of outputs in response to changes in input or analysis configuration, including diagnostic exclusion, without repeating the entire input and analysis process. 15. Pictorial representation of output, in addition to tabular representation. 16. Graphical or tabular juxtaposition of outputs with input-specific catalog data (input variants hosted by gene, causal links between diseases and gene, phenotypes annotated to disease, known modes of inheritance of disease, etc.). 17. Export of input and configuration data in a file that can be subsequently imported and modified, and from which result outputs can be regenerated. 18. Restriction to use of only human data catalogs (known direct and ontological associations between diseases, genes, and phenotypes) in differential disease diagnosis and variant rank estimation for clinical decision support. 19. Calculation of disease and variant rankings without the use of externally-computed phenotype-based rankings. 20. Deductive-reasoning-based variant ranking through inference of host gene phenotypic relevance from semantic similarities of intact diseases to which genes are causally linked

### Transitive prioritization

Our tool uses transitive prioritization to link genetic variations to phenotypic traits through differential intermediate diseases. The retention of differential intermediate diseases plays an important role in the facilitation of our visualization scheme and curatorial process: because they can be visualized, users can exclude disease alternatives deemed diagnostically irrelevant. This curation can in turn further improve the performance of transitive prioritization. Importantly, algorithms such as PhenIX [18] employ phenotypic collapsing to map genes to phenotype sets. As depicted in Fig. 1b, collapsing phenotypes across diseases can result in potentially flawed semantic scores. Our results show that transitive prioritization has better performance and retains this curatorial functionality.

### Visualization of semantic relationships

The HPO [19] is a high dimensional feature space for representing the complexity of pathologies that are observed in human disease. Representing points in this space in a low-dimensional map is a difficult computational challenge. Our attempts to use classical MDS to represent these data reveal the challenge of dimension reduction for these data. Although the global plot reveals gross features of disease relationships, the error of inter-point distances in the low-dimensional projection results in loss of semantic relationships, making it difficult to use this global projection in diagnostics. As an alternative, we developed the radar plot. This local view retains an accurate representation of the semantic similarity of differential intermediate diseases to the case’s phenotypes using distance from the proband placed at the center of the graphic. The relationships between diseases are used to construct an approximate circumferential arrangement of points. Research to explore other approaches to two-dimensional representations of semantic similarity is warranted.

### Semantic similarity

Our tool relies on semantic similarity to analyze patient indication content against genetic variation and prior knowledge. As in previous work, we employed the Resnik metric [37] in addition to alternatives. The Resnik method takes a weighted combination of lowest common ontological ancestor matches among query and target phenotypes to assign scores. A simpler approach, ATO [38], counts the unique overlap of terms, including their ontological ancestry. This simple overlap of terms performed better rank estimation in our analysis of reported human exomes (Fig. 3f). Although the Resnik similarity metric has been extensively employed in this field, our results suggest that alternative metrics should be explored, and we observe that Resnik might not be optimal in all situations. We propose that the Resnik score may suffer from certain limitations. First, to compute the similarity score, the information content of the least-commonly-annotated common ancestor phenotype across all pairs of query and disease phenotypes is averaged. This averaging can dilute or overestimate term contributions to scores for densely or sparsely phenotyped diseases because the quantities of terms annotated to each disease can vary. Second, the weighting of terms in the Resnik calculation uses information content, defined as the negative logarithm of frequency of an ontological term in the ancestry among all cataloged diseases [37]. This choice of weights may be suboptimal because of the strongly non-linear nature of the log-transformation that causes the most rare terms to have extremely high weights. Third, under Resnik, each query term makes an independent additive contribution to similarity, but the same nodes in the ontological tree may be recruited across multiple terms. Therefore, this additive approach permits the same nodes in the ontology to contribute to query scoring multiple times. This stands in contrast to the more direct overlap approach in which each phenotype contributes only once to each score [38].

### Annotation data recruitment of additional information to improve variant prioritization

The approach presented in this paper does not explicitly use deleteriousness scores based on considerations from structural biology, such as those generated via PolyPhen or SIFT [51, 52], to transitively rank variant genes. It does, however, recruit variant pathogenicity scores and frequencies, and it represents this information in displays using the color and size, respectively, of radar plot points for diseases to which the variant host genes are causally linked. We include this content as metadata in the visual display so that users can incorporate it into their curation. Future extensions that more explicitly incorporate these approaches may further improve the accuracy of variant gene prioritization. These areas present opportunities for future research.

### Disease gene discovery

An additional feature of our tool is discovery driven by the synthesis of semantic matching to the known catalog with prior gene knowledge in the form of protein interaction networks. The approach increases the likelihood of identifying possible disease-causing variants not matched to OMIM entries and of identifying novel gene-to-phenotype relationships that can be associated with existing disease genes. Our approach leverages known causal gene associations in phenotypic neighborhoods of top semantic matches to select phenotypically matched genes for discovery of candidates. This approach exploits known inter-gene relationships defined in the PINA2 PPI network [47]. Additional data sources, such as gene expression, gene annotation (GO), or transcription factor binding databases could be used to extend the power of phenotypically guided disease gene discovery [53–55]. This area also merits further inquiry.

## Conclusions

Our visual approaches represent a new scheme for variant prioritization in genome-wide diagnostics. We explored algorithmic alternatives, compared our work with other available software, and encapsulated this work in our novel tool, called OMIM Explorer. The tool is fundamentally structured around a visual map of known genetic diseases based on semantic similarity. Patient phenotype and variant information, as well as additional external information on variant class, frequency, and pathogenicity, are superimposed on this map. This approach provides visual guidance to the diagnostician or physician for evaluation. The tool also directs additional informative phenotyping, helps provide rationale for possible co-occurrence of multiple diagnoses, and facilitates the discovery of novel gene-to-phenotype associations. We validated our tool using existing catalogs of known diseases, and we evaluated performance using a previously published cohort of exome cases from the BMGL diagnostic laboratory [31]. Ultimately, this software promises to positively impact efficiency and communication between clinicians and molecular diagnostics laboratories. Our online tool and links to detailed tutorial videos of example use cases are freely available at http://www.omimexplorer.com.

## Additional files

Additional file 1: Figure S1. Signal-to-noise ratios of known disease classes in semantic space. Signal is computed as the mean semantic similarity between all pairs of diseases within a known (A) HDN or (B) OMIM Phenotypic Series class (gray bars). Noise is computed as the mean similarity between all pairs of diseases in each class *C* and those not in *C* (black line). Scores are relativized to the highest within-class average. Signal-to-noise ratios were consistently above one, indicating high accuracy in the semantic scoring process. (PDF 338 kb) Additional file 2: Figure S2. Performance of global map visual projection. Semantic space signal-to-noise ratios of known disease classes are recovered in MDS visual space. Signal is computed as the mean semantic similarity between all pairs of diseases within a known (A) HDN or (B) OMIM Phenotypic Series class (gray bars). Noise is computed as the mean similarity between all pairs of diseases in each class *C* and those not in *C* (black line). Scores are relativized to the highest within-class average. Signal-to-noise ratios were consistently above one, indicating retention of pre-MDS semantic space relationships in post-MDS visual space. (PDF 259 kb) Additional file 3: Figure S3. Validation of global map visualization via clustering of phenotype, genotype, and known disease class spectra. (A) Disease spectrum for the gene *FGFR2* (9 OMIM diseases). (B) Disease spectrum for the phenotype “Craniosynostosis” (47 OMIM diseases). Note that seven diseases with this phenotype are also in the *FGFR2* spectrum. (C) HDN class “Ophthalmological” (broad eye diseases). (D) OMIM Phenotypic Series “Night Blindness, Congenital Stationary” (specific eye diseases). (E) HDN class “Skeletal” (broad bone diseases). (F) OMIM Phenotypic Series “Epiphyseal dysplasia, multiple” (specific bone diseases). (PDF 1628 kb) Additional file 4: Figure S4. Semantic neighborhood preservation in visual space of global map. The nearest neighborhood in visual space (A) correlates positively with the nearest neighborhood in semantic space, but (B) insufficiently to facilitate visual detection of exact diagnoses from the global map via the coordinate-dependent convex combination method of projecting patients into the global map. (PDF 940 kb) Additional file 5: Figure S5. Causal variant gene prioritization in solved clinical cohort cases and semantic algorithm score comparison. OE transitively computed a median rank of 3 (top 1 %) for host genes via maximum annotated Resnik similarity score, and 2 (top 1 %) via maximum ancestral overlap. As comparator metrics to the transitive prioritization approaches, we computed scores using direct HPO term-to-gene annotations and unions of phenotypes collapsed from the all diseases associated with each gene via the OMIM Morbidmap. The cumulative distribution curve demonstrates the quality of solutions within a given rank as the percentage of the 47 cases with variant genes correctly ranked at a given threshold. We report the median because it robustly separates the top half of a sample from the bottom half. The transitive maximum ancestral overlap method achieved the lowest median rank, while the transitive maximum OMIM catalog-weighted ancestral overlap method achieved the highest median rank percentile. (PDF 9 kb) Additional file 6: Table S1. Tabular summary of comparison of relative performance. Phenotype and filtered genotype data for the 47 cohort cases with reported molecular diagnoses were analyzed via the transitive maximum OE algorithms, phenotype-collapsing alternative algorithms, and an array of comparator tools. While OE assigned to the reported variant genes median ranks of 2 to 3, the comparator tools assigned median ranks of 1.5 to 54. OE returned reported variant gene scores for more cases, and with lower median ranks, than did 4 of the 5 comparator tools. Phen-Gen did not return scores for the reported variant gene in 32 of the 47 cases (68.09 %), but outperformed OE, with a median rank of 1.5, across the 15 cases (31.91 %) for which it did return scores for the reported variant gene. (PDF 27 kb) Additional file 7: Figure S6. OE radar map performance on an individual solved clinical case study (Resnik similarity). A case (yellow triangle) with indications of sinus bradycardia, pericardial effusion, delayed central nervous system myelination, epileptic encephalopathy, gastroesophageal reflux, encephalopathy, microcephaly, intellectual disability, and seizures. The filtered exome identified candidate variation in 145 OMIM Morbidmap genes. Variants were ranked via transitive maximum unweighted ancestral term overlap similarity. (A) Top candidate diseases (TCDs) of the differential intermediate. The 500 TCDs by semantic similarity (colored circles) are represented in the radar map. The reported *SCN8A* variant [ClinVar: SCV000245399.1] present in the patient is transitively ranked at 3 via the MIM #614558 rank of 54. (B) TCDs with cataloged causal variants. The 500 TCDs are filtered to those with causal gene variants cataloged in the OMIM Morbidmap. The *SCN8A* variant is transitively ranked at 3 via the MIM #614558 rank of 42. (C) Exome-linked TCDs. The Morbidmap TCDs are filtered to 229 diseases associated with genes variant in the patient. The *SCN8A* variant is transitively ranked 3 via the MIM #614558 rank of 4. (D) Exome TCDs with mandatory phenotypes. The 229 exome TCDs are filtered to 29 known to present with intellectual disability as observed in the patient. The *SCN8A* variant is transitively ranked 1 via the MIM #614558 rank of 1. (E) Interactive curation of exome TCDs. Medical knowledge is used to rule out 16 of the 29 remaining TCDs from the differential due to absence of their hallmark features. (F) Display of the variant gene. Early infantile epileptic encephalopathy is caused by variants in *SNC8A*, which is variant in the patient. The detected variant is rare and has high pathogenicity. (G) Display of a curatorially excluded TCD. Carpenter syndrome, caused by variants in *RAB23*, is excluded because characteristic features of skull, hand, or foot abnormalities were not reported. (PDF 1629 kb)

## Abbreviations

ATO: ancestral term overlap
BCM: Baylor College of Medicine
BMGL: Baylor Miraca Genetics Laboratory
GO: Gene Ontology
HDN: Human Disease Network
HPO: Human Phenotype Ontology
MAF: minor allele frequency
MDS: multidimensional scaling
MIM: Mendelian Inheritance in Man database
OE: OMIM Explorer
OMIM: Online Mendelian Inheritance in Man (web resource)
PINA: Protein Interaction Network Analysis (platform)
PPI: protein–protein interaction
VCF: Variant Call Format (file)
