# Supplementary figures and images for: A visual and curatorial approach to clinical variant prioritization and disease gene discovery in genome-wide diagnostics
Source: Genome Med. 2016 Feb 2;8:13. doi: 10.1186/s13073-016-0261-8 (PMC4736244; doi:10.1186/s13073-016-0261-8)

**A**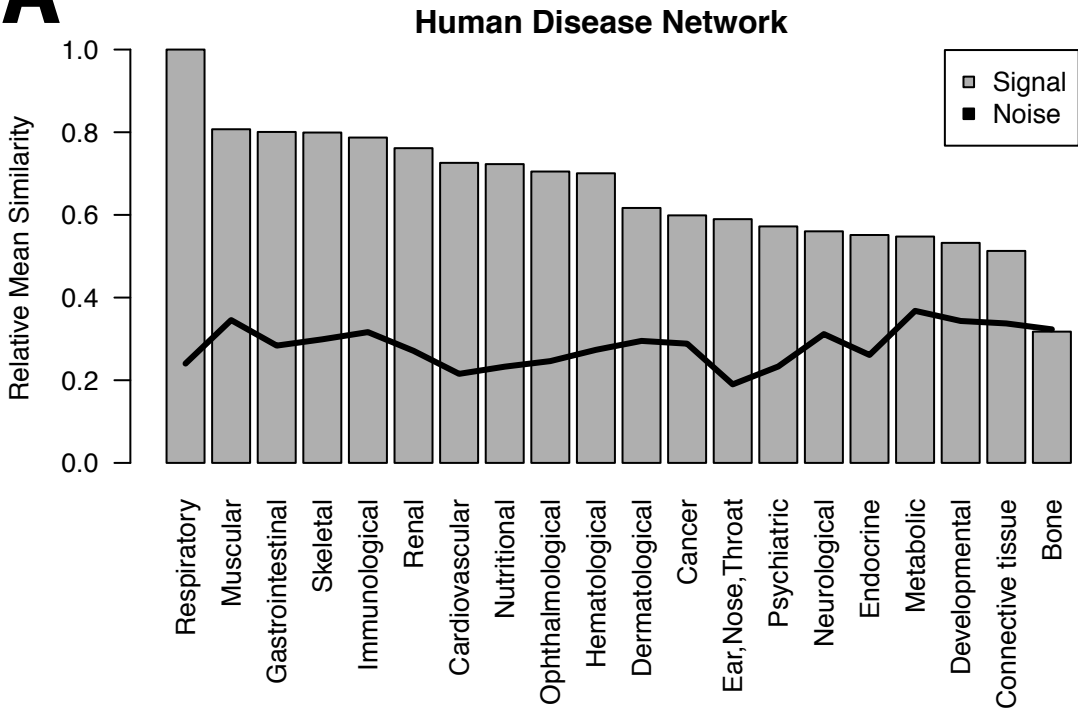**B**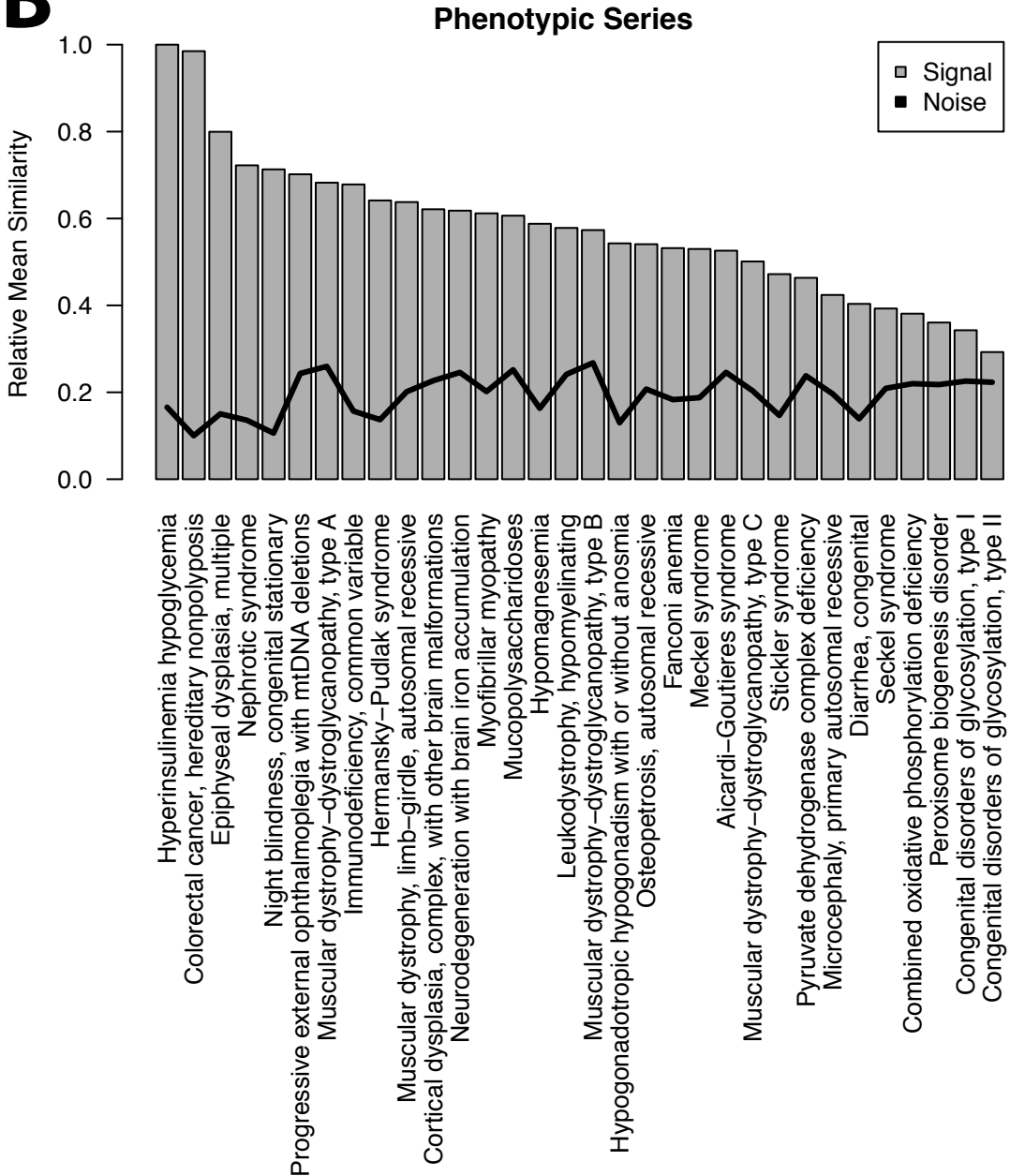

Supplement: Additional file 1: Figure S1. — Signal-to-noise ratios of known disease classes in semantic space. Signal is computed as the mean semantic similarity between all pairs of diseases within a known (A) HDN or (B) OMIM Phenotypic Series class (gray bars). Noise is computed as the mean similarity between all pairs of diseases in each class C and those not in C (black line). Scores are relativized to the highest within-class average. Signal-to-noise ratios were consistently above one, indicating high accuracy in the semantic scoring process. (PDF 338 kb) [file 13073_2016_261_MOESM1_ESM.pdf]

# A

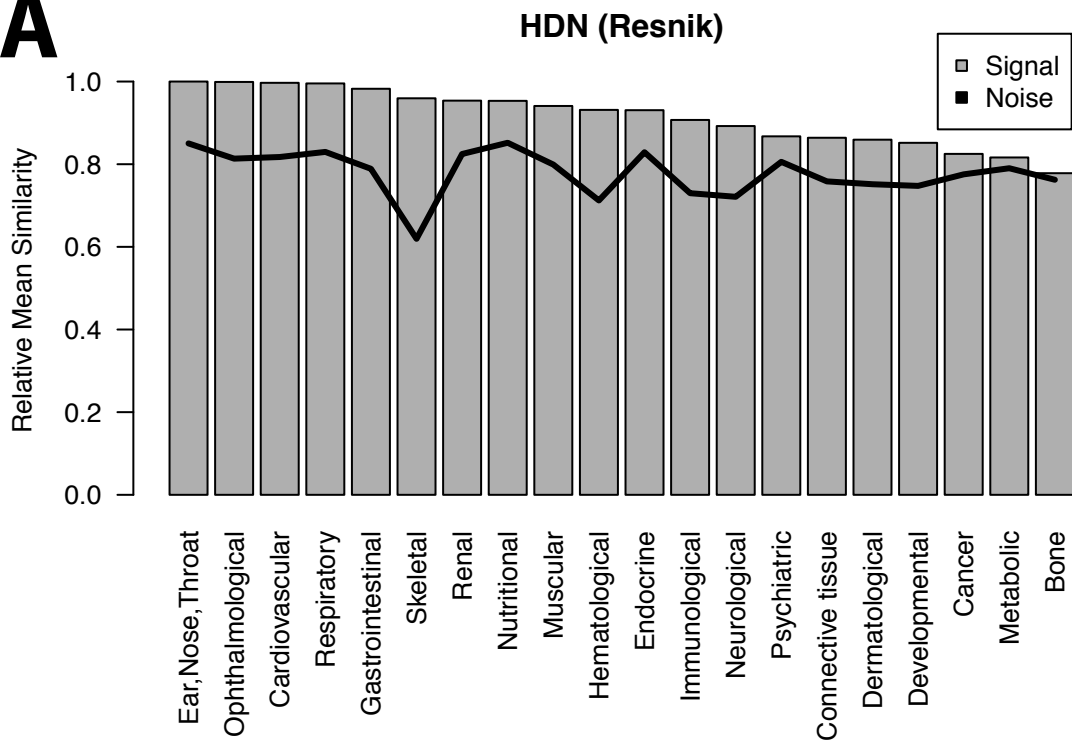

# B

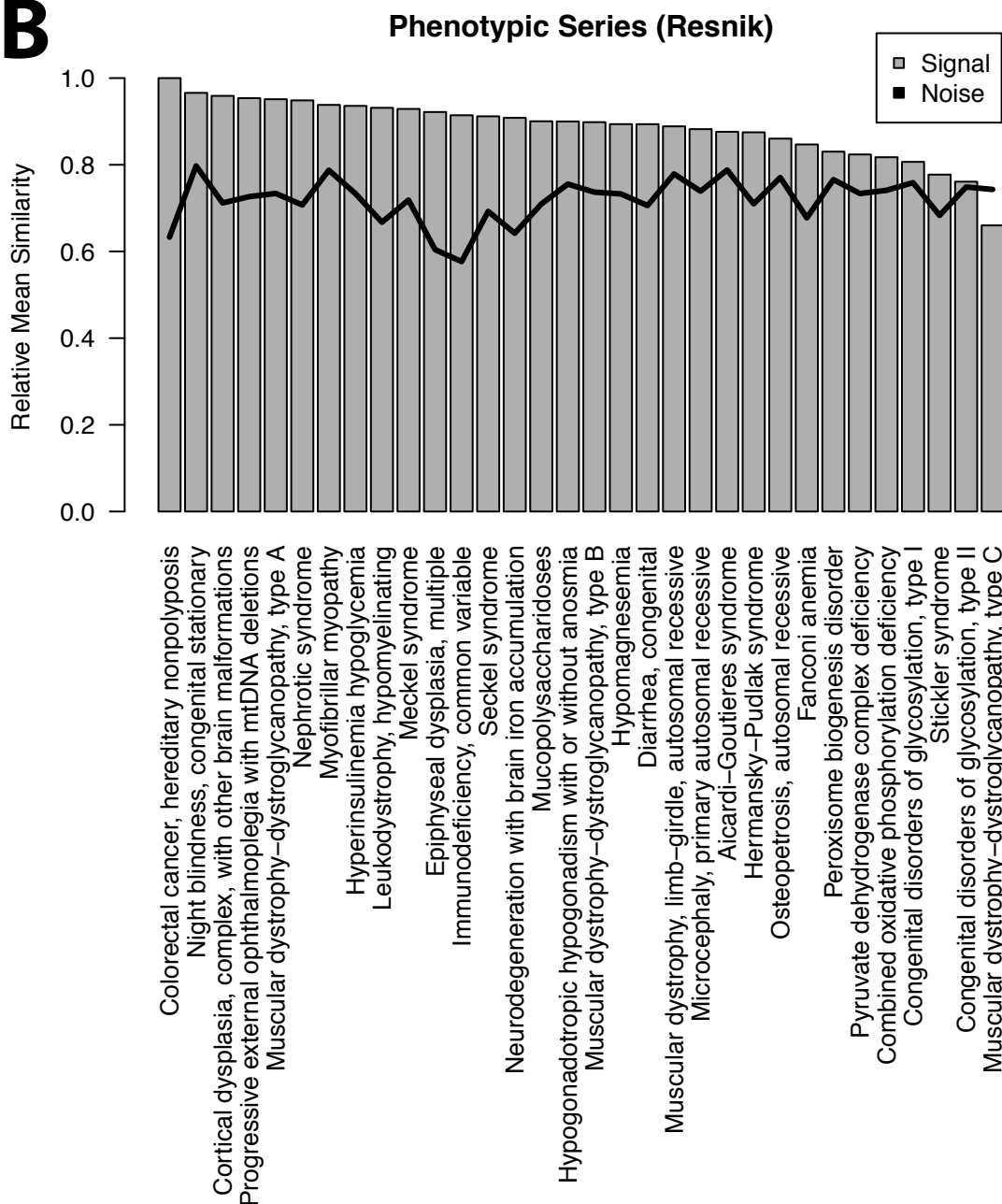

Supplement: Additional file 2: Figure S2. — Performance of global map visual projection. Semantic space signal-to-noise ratios of known disease classes are recovered in MDS visual space. Signal is computed as the mean semantic similarity between all pairs of diseases within a known (A) HDN or (B) OMIM Phenotypic Series class (gray bars). Noise is computed as the mean similarity between all pairs of diseases in each class C and those not in C (black line). Scores are relativized to the highest within-class average. Signal-to-noise ratios were consistently above one, indicating retention of pre-MDS semantic space relationships in post-MDS visual space. (PDF 259 kb) [file 13073_2016_261_MOESM2_ESM.pdf]

**A**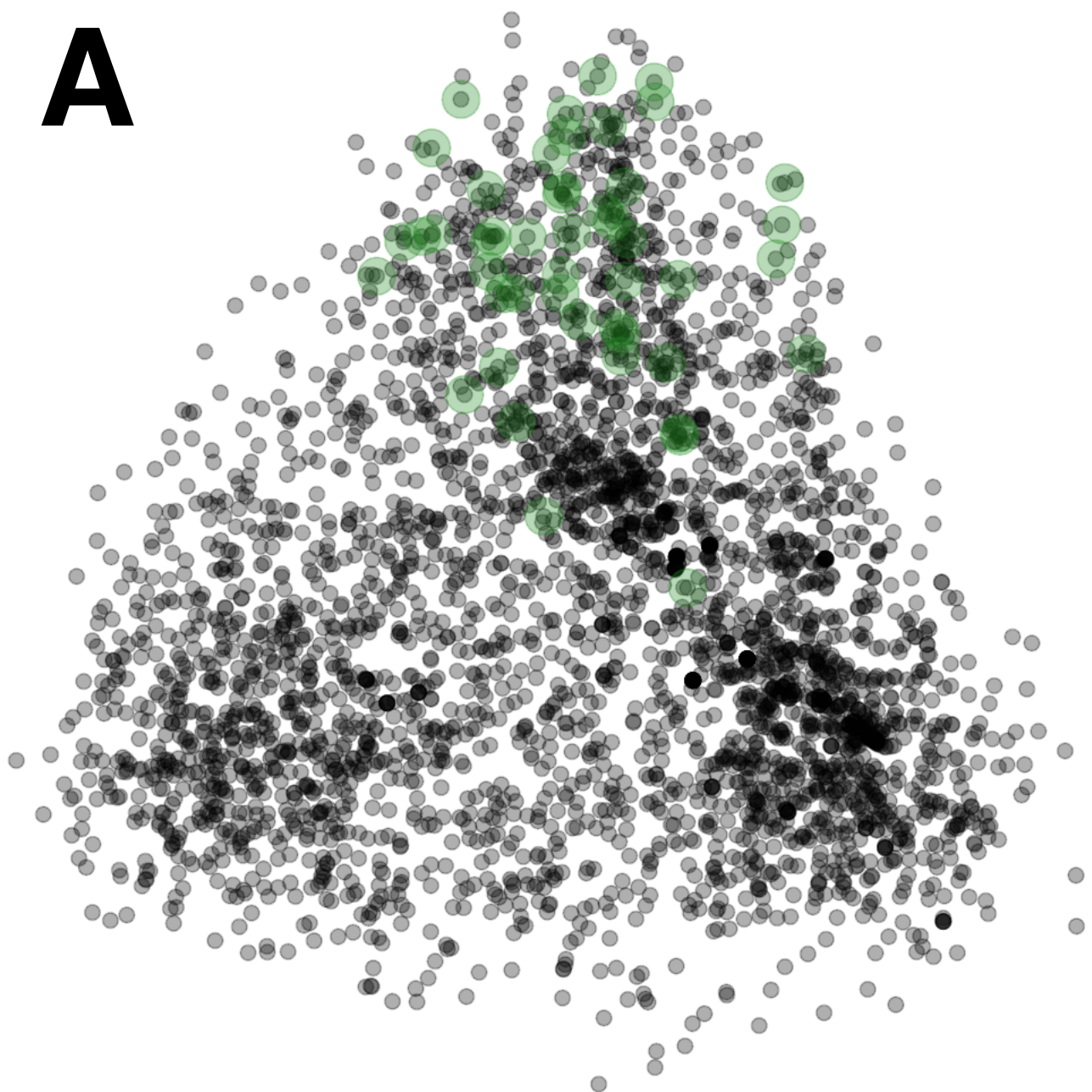**B**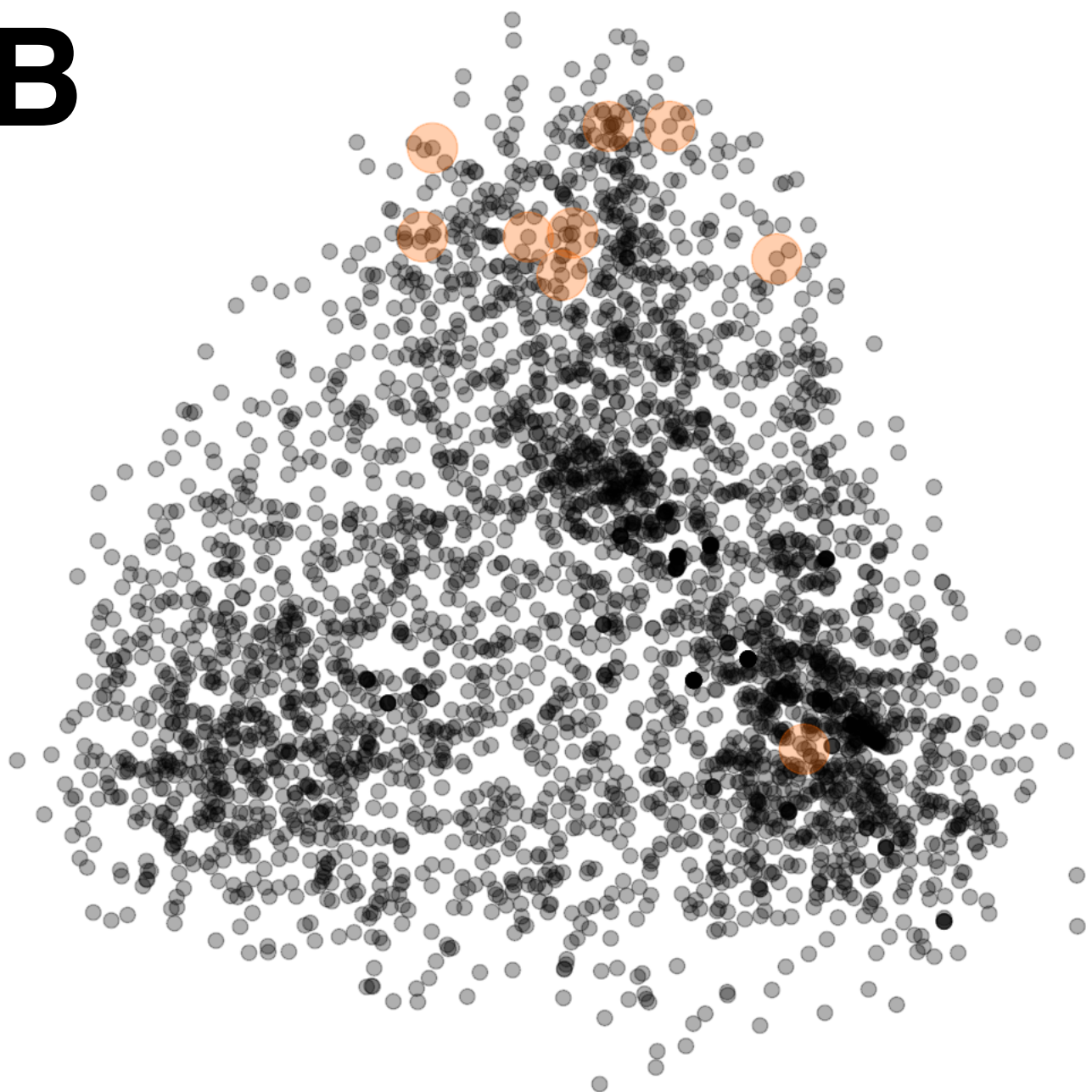**C**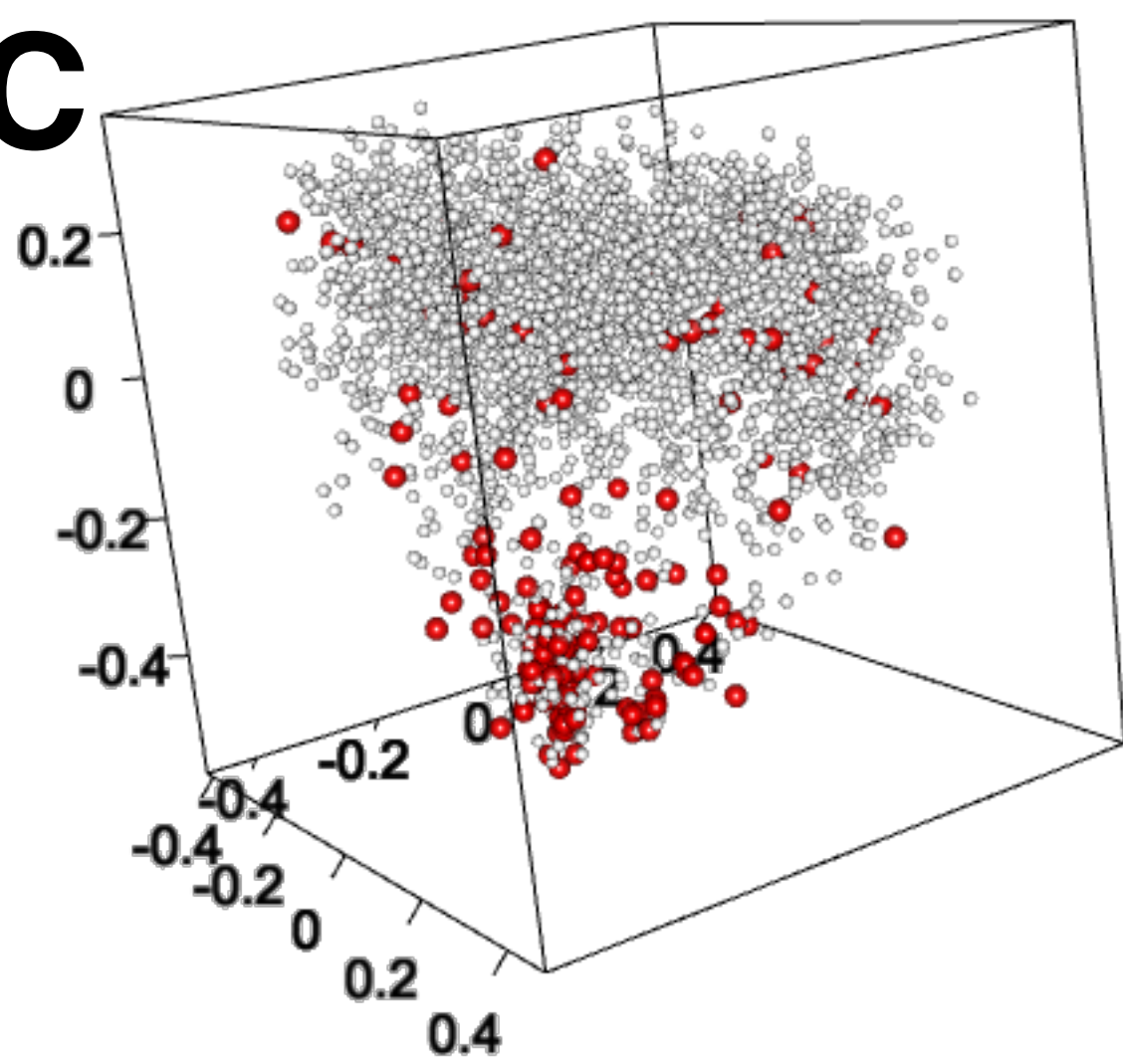**D**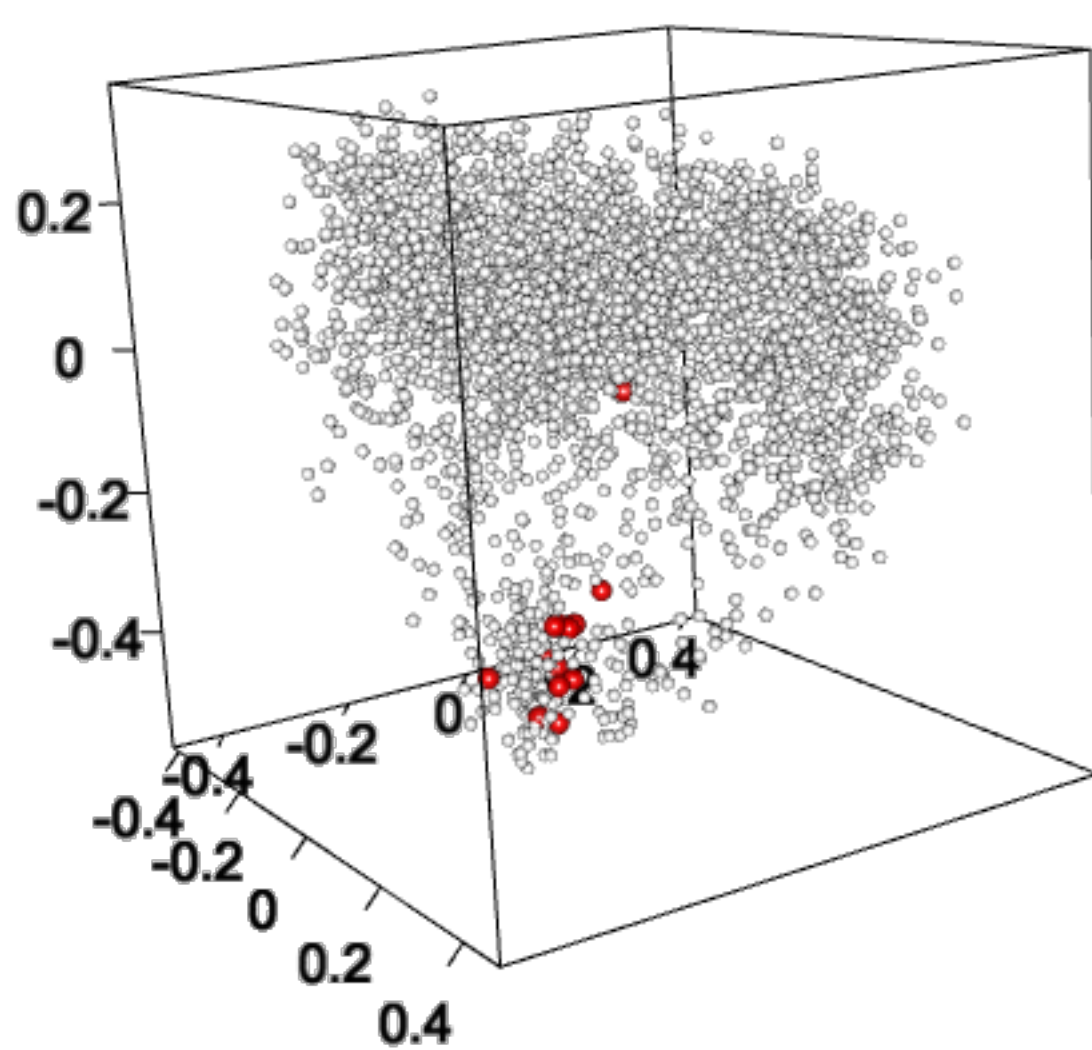**E**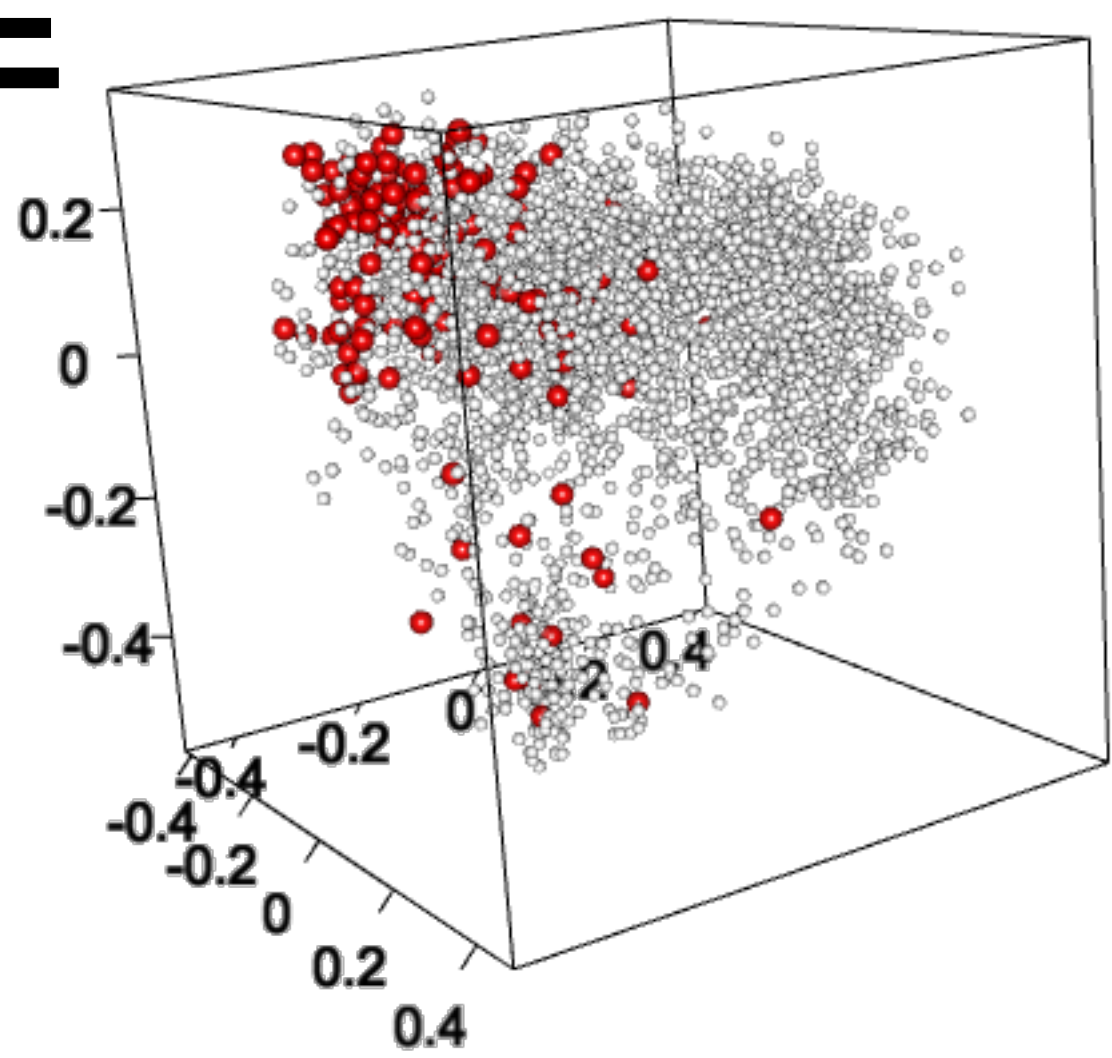**F**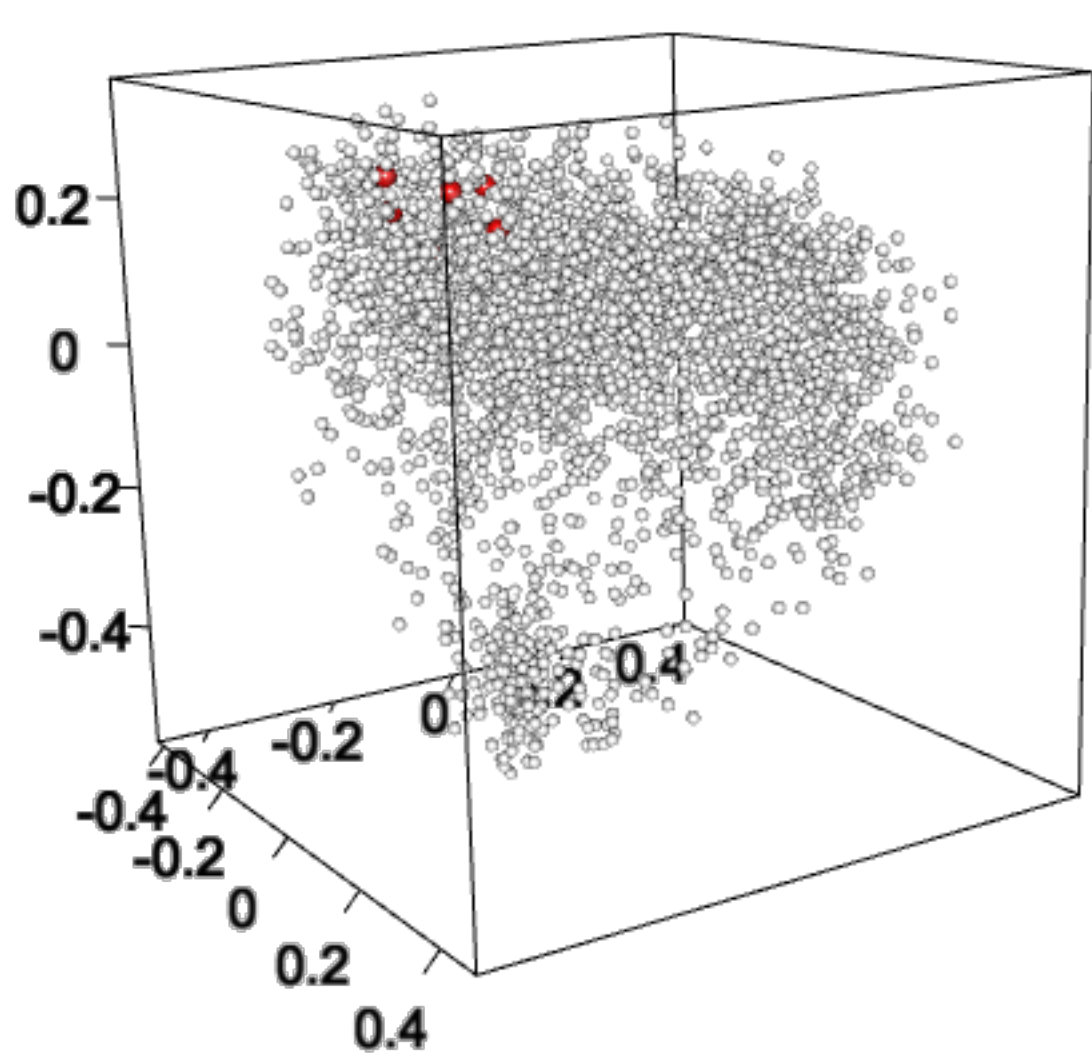

Supplement: Additional file 3: Figure S3. — Validation of global map visualization via clustering of phenotype, genotype, and known disease class spectra. (A) Disease spectrum for the gene FGFR2 (9 OMIM diseases). (B) Disease spectrum for the phenotype “Craniosynostosis” (47 OMIM diseases). Note that seven diseases with this phenotype are also in the FGFR2 spectrum. (C) HDN class “Ophthalmological” (broad eye diseases). (D) OMIM Phenotypic Series “Night Blindness, Congenital Stationary” (specific eye diseases). (E) HDN class “Skeletal” (broad bone diseases). (F) OMIM Phenotypic Series “Epiphyseal dysplasia, multiple” (specific bone diseases). (PDF 1628 kb) [file 13073_2016_261_MOESM3_ESM.pdf]

# Ranks of Genes with Reported Causal Variants (n=47)

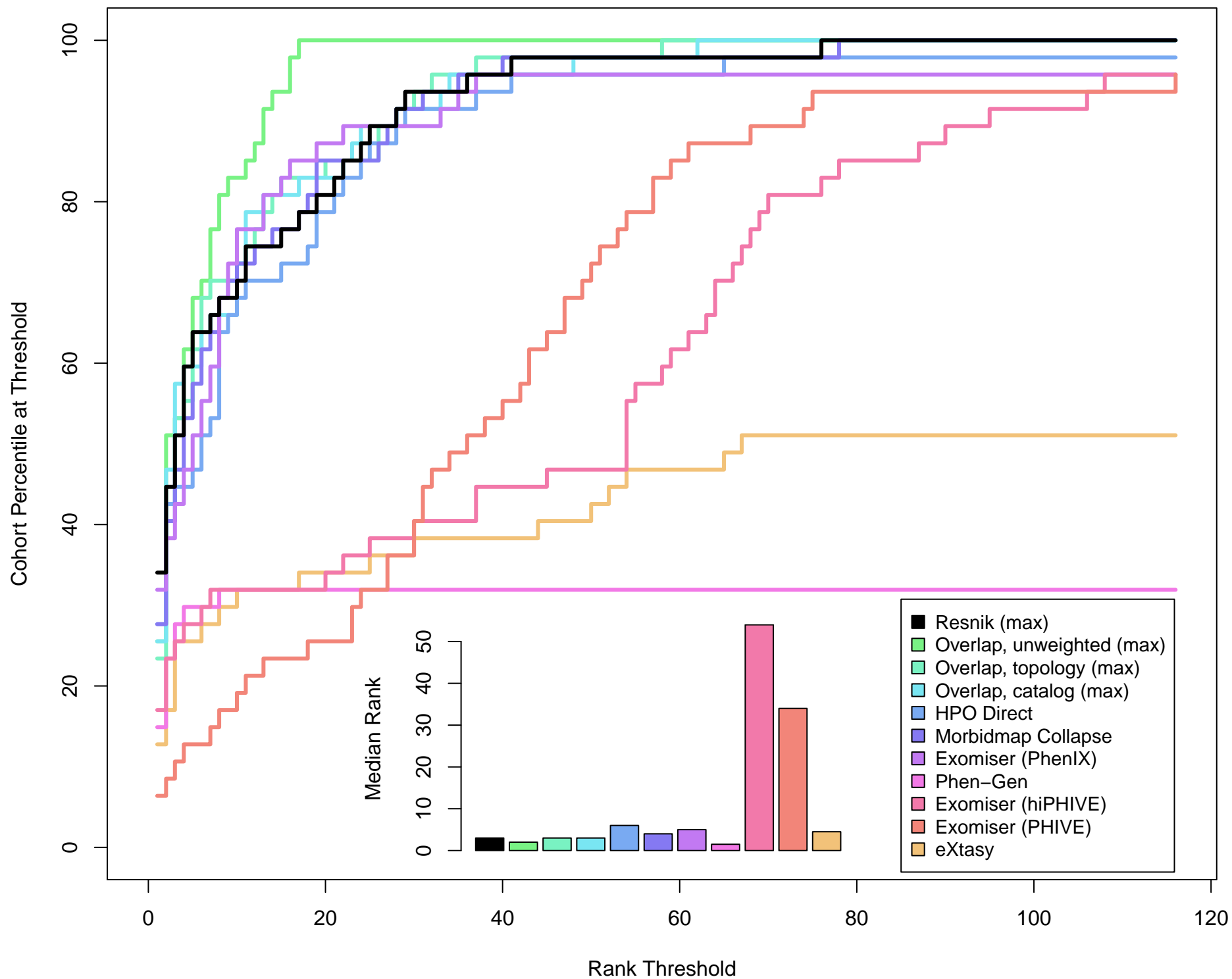

Supplement: Additional file 5: Figure S5. — Causal variant gene prioritization in solved clinical cohort cases and semantic algorithm score comparison. OE transitively computed a median rank of 3 (top 1 %) for host genes via maximum annotated Resnik similarity score, and 2 (top 1 %) via maximum ancestral overlap. As comparator metrics to the transitive prioritization approaches, we computed scores using direct HPO term-to-gene annotations and unions of phenotypes collapsed from the all diseases associated with each gene via the OMIM Morbidmap. The cumulative distribution curve demonstrates the quality of solutions within a given rank as the percentage of the 47 cases with variant genes correctly ranked at a given threshold. We report the median because it robustly separates the top half of a sample from the bottom half. The transitive maximum ancestral overlap method achieved the lowest median rank, while the transitive maximum OMIM catalog-weighted ancestral overlap method achieved the highest median rank percentile. (PDF 9 kb) [file 13073_2016_261_MOESM5_ESM.pdf]
