# Supplementary material for: A visual and curatorial approach to clinical variant prioritization and disease gene discovery in genome-wide diagnostics
Source: Genome Med. 2016 Feb 2;8:13. doi: 10.1186/s13073-016-0261-8 (PMC4736244; doi:10.1186/s13073-016-0261-8)
Supplement: Additional file 4: Figure S4. — Semantic neighborhood preservation in visual space of global map. The nearest neighborhood in visual space (A) correlates positively with the nearest neighborhood in semantic space, but (B) insufficiently to facilitate visual detection of exact diagnoses from the global map via the coordinate-dependent convex combination method of projecting patients into the global map. (PDF 940 kb) [file 13073_2016_261_MOESM4_ESM.pdf]

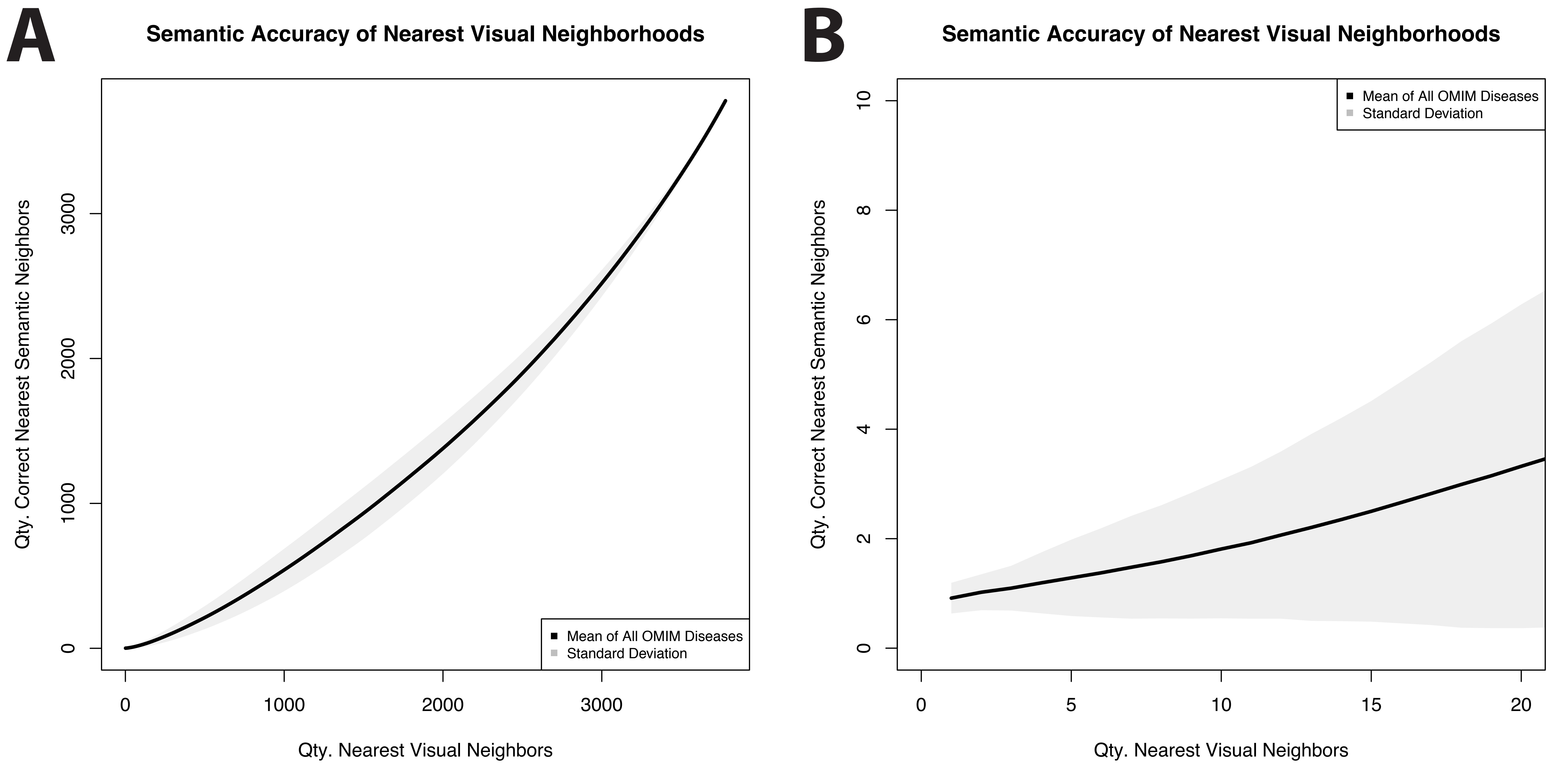

A
Semantic Accuracy of Nearest Visual Neighborhoods
Qty. Correct Nearest Semantic Neighbors
Qty. Nearest Visual Neighbors
0
1000
2000
3000
Mean of All OMIM Diseases
Standard Deviation
B
Semantic Accuracy of Nearest Visual Neighborhoods
Qty. Correct Nearest Semantic Neighbors
Qty. Nearest Visual Neighbors
0
2
4
6
8
10
5
15
20
Mean of All OMIM Diseases
Standard Deviation
