# Supplementary material for: A visual and curatorial approach to clinical variant prioritization and disease gene discovery in genome-wide diagnostics
Source: Genome Med. 2016 Feb 2;8:13. doi: 10.1186/s13073-016-0261-8 (PMC4736244; doi:10.1186/s13073-016-0261-8)
Supplement: Additional file 6: Table S1. — Tabular summary of comparison of relative performance. Phenotype and filtered genotype data for the 47 cohort cases with reported molecular diagnoses were analyzed via the transitive maximum OE algorithms, phenotype-collapsing alternative algorithms, and an array of comparator tools. While OE assigned to the reported variant genes median ranks of 2 to 3, the comparator tools assigned median ranks of 1.5 to 54. OE returned reported variant gene scores for more cases, and with lower median ranks, than did 4 of the 5 comparator tools. Phen-Gen did not return scores for the reported variant gene in 32 of the 47 cases (68.09 %), but outperformed OE, with a median rank of 1.5, across the 15 cases (31.91 %) for which it did return scores for the reported variant gene. (PDF 27 kb) [file 13073_2016_261_MOESM6_ESM.pdf]

	Algorithm	Qty. Reported Variants Scored	% of Reported Variants Scored	Median Rank of Scored Reported Variants	Mean Rank of Scored Reported Variants
**OMIM Explorer**	Resnik*	47	100	3	9.78
	Overlap, unweighted*	47	100	2	4.95
	Overlap, topology*	47	100	3	8.91
	Overlap, catalog*	47	100	3	9.28
**Alternative Methods**	HPO Direct (Phenomantics)	46	97.87	6	10.55
	Morbidmap Collapse	47	100	4	10.05
**Comparator Tools**	PhenIX**	45	95.74	5	7.62
	Phen-Gen	15	31.91	1.5	2.13
	hiPHIVE**	45	95.74	54	41.53
	PHIVE**	45	95.74	34	35.44
	eXtasy	24	51.06	4.5	18.73

**As implemented via the transitive maximum algorithm*
***As implemented by Exomiser*
