# Supplementary material for: A visual and curatorial approach to clinical variant prioritization and disease gene discovery in genome-wide diagnostics
Source: Genome Med. 2016 Feb 2;8:13. doi: 10.1186/s13073-016-0261-8 (PMC4736244; doi:10.1186/s13073-016-0261-8)
Supplement: Additional file 7: Figure S6. — OE radar map performance on an individual solved clinical case study (Resnik similarity). A case (yellow triangle) with indications of sinus bradycardia, pericardial effusion, delayed central nervous system myelination, epileptic encephalopathy, gastroesophageal reflux, encephalopathy, microcephaly, intellectual disability, and seizures. The filtered exome identified candidate variation in 145 OMIM Morbidmap genes. Variants were ranked via transitive maximum unweighted ancestral term overlap similarity. (A) Top candidate diseases (TCDs) of the differential intermediate. The 500 TCDs by semantic similarity (colored circles) are represented in the radar map. The reported SCN8A variant [ClinVar: SCV000245399.1] present in the patient is transitively ranked at 3 via the MIM #614558 rank of 54. (B) TCDs with cataloged causal variants. The 500 TCDs are filtered to those with causal gene variants cataloged in the OMIM Morbidmap. The SCN8A variant is transitively ranked at 3 via the MIM #614558 rank of 42. (C) Exome-linked TCDs. The Morbidmap TCDs are filtered to 229 diseases associated with genes variant in the patient. The SCN8A variant is transitively ranked 3 via the MIM #614558 rank of 4. (D) Exome TCDs with mandatory phenotypes. The 229 exome TCDs are filtered to 29 known to present with intellectual disability as observed in the patient. The SCN8A variant is transitively ranked 1 via the MIM #614558 rank of 1. (E) Interactive curation of exome TCDs. Medical knowledge is used to rule out 16 of the 29 remaining TCDs from the differential due to absence of their hallmark features. (F) Display of the variant gene. Early infantile epileptic encephalopathy is caused by variants in SNC8A, which is variant in the patient. The detected variant is rare and has high pathogenicity. (G) Display of a curatorially excluded TCD. Carpenter syndrome, caused by variants in RAB23, is excluded because characteristic features of skull, hand, or foot abnormalities were not reported. (PDF 1 [file 13073_2016_261_MOESM7_ESM.pdf]

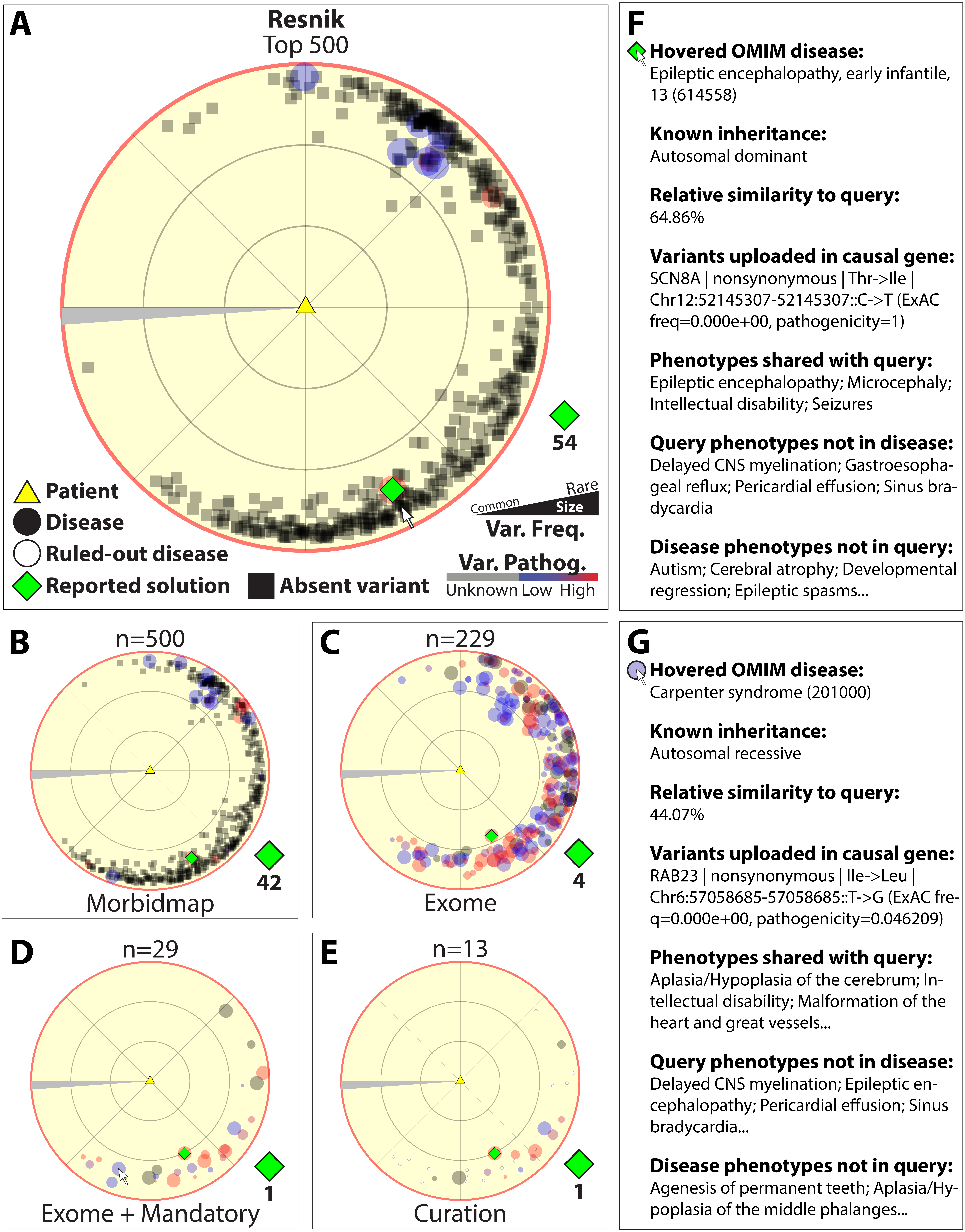
A
Resnik
Top 500
54
Patient
Disease
Ruled-out disease
Reported solution
Absent variant
Common
Rare
Size
Var. Freq.
Var. Pathog.
Unknown Low High
B
n=500
42
Morbidmap
C
n=229
4
Exome
D
n=29
1
Exome + Mandatory
E
n=13
1
Curation
F
Hovered OMIM disease:
Epileptic encephalopathy, early infantile, 13 (614558)
Known inheritance:
Autosomal dominant
Relative similarity to query:
64.86%
Variants uploaded in causal gene:
SCN8A | nonsynonymous | Thr->Ile | Chr12:52145307-52145307::C->T (ExAC freq=0.000e+00, pathogenicity=1)
Phenotypes shared with query:
Epileptic encephalopathy; Microcephaly; Intellectual disability; Seizures
Query phenotypes not in disease:
Delayed CNS myelination; Gastroesophageal reflux; Pericardial effusion; Sinus bradycardia
Disease phenotypes not in query:
Autism; Cerebral atrophy; Developmental regression; Epileptic spasms...
G
Hovered OMIM disease:
Carpenter syndrome (201000)
Known inheritance:
Autosomal recessive
Relative similarity to query:
44.07%
Variants uploaded in causal gene:
RAB23 | nonsynonymous | Ile->Leu | Chr6:57058685-57058685::T->G (ExAC freq=0.000e+00, pathogenicity=0.046209)
Phenotypes shared with query:
Aplasia/Hypoplasia of the cerebrum; Intellectual disability; Malformation of the heart and great vessels...
Query phenotypes not in disease:
Delayed CNS myelination; Epileptic encephalopathy; Pericardial effusion; Sinus bradycardia...
Disease phenotypes not in query:
Agenesis of permanent teeth; Aplasia/Hypoplasia of the middle phalanges...
